# Endophytic Fungi of *Calea pinnatifida* (Asteraceae): Dereplication of Crude Extracts, Antimicrobial Properties, and Identification of New Tetronic Acid Derivative Produced by *Hypomontagnella barbarensis*

**DOI:** 10.3390/jof11010022

**Published:** 2024-12-31

**Authors:** Bianca Barna, Lhaís Araújo Caldas, Jackson Monteiro, Augusto Leonardo dos Santos, Renata Castiglioni Pascon, Marcelo Afonso Vallim, Marcelo José Pena Ferreira, Sarah Santos Gonçalves, Glaucia Queiroz dos Santos, Anderson Messias Rodrigues, Jamile Ambrósio de Carvalho, Suzan Pantaroto de Vasconcellos, Patricia Sartorelli

**Affiliations:** 1 Institute of Environmental, Chemical and Pharmaceutical Sciences, Federal University of São Paulo, Diadema 09972-270, SP, Brazil; bianca.barna@unifesp.br (B.B.); lhais.caldas@unifesp.br (L.A.C.); jackson.monteiro@unifesp.br (J.M.); augusto.leonardo@unifesp.br (A.L.d.S.); renata.pascon@gmail.com (R.C.P.); marcelo.vallim@unifesp.br (M.A.V.); suzan.pantaroto@unifesp.br (S.P.d.V.); 2 Department of Botany, Institute of Biosciences, University of São Paulo, São Paulo 05508-090, SP, Brazil; marcelopena@ib.usp.br; 3 Department of Pathology, Federal University of Espírito Santo, Goiabeiras, Vitória 29075-910, ES, Brazil; sarah.tavares@ufes.br (S.S.G.); glaucia.santos@ufes.br (G.Q.d.S.); 4 Department of Microbiology, Immunology and Parasitology, Federal University of São Paulo, São Paulo 04023-062, SP, Brazil; amrodrigues.amr@gmail.com (A.M.R.); jamileambrosio@hotmail.com (J.A.d.C.)

**Keywords:** *Calea pinnatifida*, endophytic fungi, *Hypomontagnella barbarensis*, antimicrobial activity, dereplication

## Abstract

Endophytic fungi are increasingly being recognized for their diverse metabolites that may exhibit antimicrobial properties. In our study, we isolated seven endophytic fungal strains from *Calea pinnatifida*, which were identified as *Hypomontagnella barbarensis*, *Neopestalotiopsis clavispora*, *Nigrospora sacchari-officinarum*, *Annulohypoxylon moriforme*, *Colletotrichum siamense*, and *Colletotrichum karstii* (with two isolates from the same species). Furthermore, the antimicrobial activity of the extracts was assessed, revealing that the extract from *Hypomontagnella barbarensis* demonstrated activity against *Staphylococcus aureus*. Further investigation of secondary metabolites, employing UHPLC-HR-ESI-MS/MS in combination with molecular networking, facilitated annotation of the nine compounds. Of these, five were identified based on matches with the GNPS spectral library, and four were predicted based on the molecular network. Notably, from the extract of *Hypomontagnella barbarensis*, two pairs of diastereoisomeric acyl-tetronic acid derivatives were isolated and characterized using MS and NMR spectroscopy. This study highlights the potential of endophytic fungi as a valuable source of novel antimicrobial agents.

## 1. Introduction

The search for new antimicrobial compounds has emerged as a promising alternative to treatments for diseases caused by microorganisms such as fungi and bacteria, with the majority of antibiotic compounds being isolated from natural products. Thus, these bioactive compounds have proven to be of great interest to researchers, particularly given the growing concern regarding microbial resistance to commonly used treatments, which poses a global health threat [1]. Modern medicine, which relies heavily on effective antimicrobial drugs, faces significant challenges with these emerging demands [2,3]. Among the primary causes of bacterial infections in humans is *Staphylococcus aureus*, a major health concern in both hospital and community settings [4]. Additionally, some strains of *Saccharomyces cerevisiae* have been responsible for invasive infections more frequently in recent years in patients with underlying diseases with opportunistic infections, mainly in immunodependent individuals [5,6]. As a well-established model organism, this yeast species is widely used to evaluate the biological activities of substances. In addition, candidiasis, caused by *Candida* sp., is frequently associated with congenital or acquired immunodeficiencies, neoplastic and degenerative diseases, and immunosuppression [4,7]. Thus, in response to infections, over the last century, research has been dedicated to discovering and applying bioactive compounds from various sources as a strategy to solve the problem [8].

The use of natural products from fungi, including terpenes, peptides, and mainly polyketides, has demonstrated properties that can be effective against bacteria and fungi and has emerged as an alternative to treatments [8,9,10,11,12,13,14,15]. For instance, compounds such as 6-O-methylalaternin and altersolanol A, isolated from *Ampelomyces* sp. (from *Urospermum picroides*, Asteraceae), have exhibited antimicrobial activity against Gram-positive pathogens, including *Staphylococcus aureus*, *S. epidermidis*, and *Enterococcus faecalis* [16]. Antimicrobial metabolites have also been described from the extract of the endophyte *Nodulisporium* sp., isolated from the plant *Erica arborea* (Ericaceae), including nodulisporins D–F with antibacterial and fungicidal properties [17].

Asteraceae, notable for its medicinal applications, comprises approximately 33,000 species grouped into 1911 genera [18], some of which harbor endophytic microorganisms [11]. Although there is great diversity and abundance of Asteraceae species, only a small percentage of endophytic fungi have been studied. According to Caruso et al. [11], research carried out on the endophytes of some Asteraceae species has shown interesting compounds, such as isofusidiene I, A, B, C, and D from endophytes of the genus *Chalara* associated with *Artemisia vulgaris*, which exhibit both antibacterial and antifungal activity, ref. [19], and phomosin K with antimicrobial activity from *Phomopsis* species inhabiting the internal tissues of *Notobasis syriaca* [20].

In this context, *Calea pinnatifida* has emerged as a plant with remarkable pharmacological potential due to its traditional medicinal properties, including use in folk medicine as an antimalarial, analgesic, anti-inflammatory, and for the treatment of gastrointestinal diseases [21]. However, despite their relevance in traditional medicine, endophytic fungi associated with *C. pinnatifida* remain largely unexplored. Therefore, this research gap offers a unique opportunity to investigate and identify bioactive compounds with pharmaceutical potential [22,23]. Therefore, this study investigated endophytic fungi associated with *C. pinnatifida* to identify new antimicrobial compounds. To achieve this, the analysis of antimicrobial extracts was carried out by UHPLC-HRMS-MS combined with molecular networking. This strategy led to selecting one endophytic fungus whose extract allowed the isolation of two pairs of diastereoisomeric tetronic acid derivatives, including the novel compound Noduslifuranol.

## 2. Materials and Methods

### 2.1. General Experimental Procedures

^1^H and ^13^C NMR spectra were recorded at 300 and 75 MHz, respectively, on an Ultrashield 300 Avance III spectrometer (Bruker-BioSpin, Ettlingen, Germany). It was equipped with a 5 mm trinuclear inverse detection probe with z-gradient (TXI). The temperature was controlled by a BCU I accessory at 25 °C. Deuterated methanol (MeOD-*d_4_*) (Sigma-Aldrich, St. Louis, MO, USA) was used as the solvent and internal standard. High-Performance Liquid Chromatography with Diode Array Detection (HPLC-DAD) analysis was carried out on an Ultimate 3000 liquid chromatograph with a UV-DAD detector (Dionex, Sunnyvale, CA, USA), using a Kinetex C_18_ analytical column (Core-Shell—4.6 μm-150.0 mm × 4.6 mm) for method standardization and chemical profile analysis and a Kinetex column C_18_ semi-preparative column (Core-Shell—4.6 μm-150.0 mm × 10.0 mm) for compound isolation. The analyses by UHPLC-HR-MS/MS were performed in a mass spectrometer Q-ToF maxis 3G (Bruker–Daltonics, Billerica, MA, USA), equipped with an electrospray ionization source (ESI) operating with a resolution of 60,000 FWHM in positive mode with detection in a range of masses from *m*/*z* 50 to 1500 Da. The capillary voltage was fixed at 4500 V and the nozzle at 500 V. The conditions applied for the drying gas (N_2_) were 8.0 mL min^−1^, pressure 4.0 Bar, and temperature 200 °C for the flow of 0.3 mL min^−1^. UV-Vis detection (PDA) was performed at a rate of 4.6 Hz in a spectral window from 200 to 700 nm [24]. Calibration was set at less than 2 ppm using sodium formate.

### 2.2. Plant Material and Procedure of Isolation of Endophytic Fungi

Branches and leaves of *C. pinnatifida* were collected at the Botanical Garden of São Paulo—SP (coordinates 23 38′29.47″ S, 46 37′15.83″ W). The specimen (Ferreira, M. J. P. 27) has been deposited in the SPF Herbarium of the Botany Department from the Biosciences Institute of the University of São Paulo. The methodology used to isolate endophytic fungi was based on the procedure described by Santos [25]. Briefly, fresh leaves were subjected to external disinfection to remove dirt and epiphytic microorganisms. First, the leaves were immersed in 70% ethanol, 5% sodium hypochlorite, or 70% ethanol for 3, 5, or 1 min. Subsequently, the leaves were washed with sterile water to remove any alcohol or hypochlorite residue inside a laminar flow hood (Pachane, class Pa40, vertical flow). Next, fragments approximately 5 mm^2^ in size were cut and transferred to Petri dishes containing potato dextrose agar (PDA) medium with ampicillin (100 mg·L^−1^) to prevent bacterial growth. These plates were sealed and incubated at 25 °C for fungal growth. After 3 days, the grown fungi were transferred to new Petri dishes for further isolation.

### 2.3. Identification of Endophytic Fungi

Total DNA was obtained from 2 day-old monosporic colonies at 37 °C on yeast extract sucrose agar (YES) (10 g yeast extract, 75 g sucrose, 10 g agar, and 500 mL distilled water) using the PrepMan Ultra kit (Applied Biosystems, Carlsbad, CA, USA), according to the manufacturer’s recommendations. For accurate species identification, the following were sequenced: (i) fragments of internal transcribed spacer (ITS) of ribosomal DNA region using the primer pairs ITS1 and ITS4, and fragments of tubulin gene (*TUB2*) using the primer primers Bt-2a and Bt-2b. The amplification reactions were performed in a final volume of 25 µL containing 1 µL DNA (40 ng), 12.5 µL 2 PCR Master Mix (Promega Corporation, Madison, WI, USA) containing 3 mM MgCl_2_, 400 mM each dNTP, 50 U/mL of Taq Polymerase, 9.5 µL Milli-Q water, and 1 µL of each primer. The amplification protocol included an initial denaturation at 94 °C for 5 min, followed by 35 cycles of denaturation at 94 °C for 30 s, annealing for 30 s (at 52 °C and 55 °C for ITS and *TUB2*, respectively), and a final extension for 10 min at 72 °C. Amplicons were purified using a Gel and PCR Clean-Up Kit (Promega Corporation, Madison, WI, USA), according to the manufacturer’s recommendations. All cycling reactions were performed in an Eppendorf Mastercycler X50 (Eppendorf Norge AS, Oslo, Norway). The amplified fragments were sequenced in both directions using the BigDye™ Terminator v3.1 Cycle Sequencing Kit (Applied Biosystems, Foster City, CA, USA). The samples were run on a SeqStudio Genetic Analyzer (Applied Biosystems, Foster City, CA, USA) at the Laboratory of Emerging Fungal Pathogens (Federal University of São Paulo, São Paulo, Brazil) under previously described conditions [26].

The obtained sequences were assembled and edited using the DNA sequence assembly software Sequencher 4.1.4 (Gene Codes Corporation, Ann Arbor, MI, USA). Successful assembly of contigs required a minimum match percentage of ≥85 and a minimum overlap of 20. Consensus sequences were compared with sequences deposited in the public genomic databases GenBank “www.ncbi.nlm.nih.gov/genbank/ (accessed on 28 May 2024)” and Mycobank “www.mycobank.org/Pairwise_alignment (accessed on 28 May 2024)” using the BLASTn tool. We considered the parameters of E-value < 10^−5^ and identity ≥ 99% for accurate species identification.

To determine the phylogenetic distance between strains in the same family, phylogenetic trees were constructed. Thus, ITS sequences from the fungi were aligned with MAFFT “https://mafft.cbrc.jp/alignment/server/index.html (accessed on 11 March 2024)” [27] and visualized with AliViewer 1.2 Poorly aligned regions were removed using trimAI v1.2 (parameter—automated1) [28]. A maximum likelihood tree was constructed using the ITS sequences from the samples (CPFF12, CPFF14, CPFF16, CPFF41, CPFF42, and CPFF52) and ITS sequences from similar related genera. *Amanita muscaria* ITS sequence was used as an outlier (AB015700.1). The tree was built using IQ-TREE v.2 [29] with a model finder (parameters–bb 1000–bnni-nt AUTO). The resulting trees were visualized using iTOL “https://itol.embl.de/ (accessed on 11 March 2024)” [30].

For morphological characterization, the isolates were incubated on PDA at 25 °C for 7 to 15 days to evaluate their viability and purity. For genus identification, each isolate was inoculated on PDA, Oatmeal agar (OA), and Synthetic nutrient agar (SNA) [31] at 25 °C for 14 days. Macromorphology features such as colony color, diffused pigment, and texture were observed. The vegetative and reproductive mycelium characteristics, including hyphae, conidia, and conidiogenous cells, were examined. Micromorphological observations were carried out using a light microscope Leica^®^ DM500, (Leica, Wetzlar, Germany) on mounts prepared in lactophenol [32,33].

The isolated fungi were preserved using the Castellani method [34], which involves storing microorganisms in sterilized water or saline solution [35] under refrigeration (4 °C). This technique is preferably applied to young cultures to reduce metabolism and, consequently, the latency of the cells in the face of restricted nutritional sources [36], guaranteeing the preservation of the original characteristics of the culture for long periods and promoting the absence of contamination [37,38].

### 2.4. Cultivation and Extraction of Endophytic Fungi

From the fungi isolated on agar plates, fragments of cultivated mycelia were inoculated in a 150 mL Erlenmeyer flask with 70 mL of potato dextrose broth (PDB) (Becton Dickinson, São Paulo, Brazil) and kept under stirring for 7 days in an incubator (28 °C) to obtain a seed culture for solid medium inoculation. An aliquot of 1 mL of seed culture was inoculated into the rice medium in a 250 mL Erlenmeyer flask containing 50 g of autoclaved rice grains and 90 mL of water. This culture remained in static medium for 28 days in an incubator at 28 °C [39].

After cultivation, the fungi were inactivated, and the culture media inoculated with the fungi were extracted with 50 mL of ethyl acetate. The extraction process was carried out on a shaker at 120 rpm for 24 h, followed by two additional extraction cycles. The combined extract, totaling 150 mL, was then vacuum filtered. The filtrate was concentrated using a rotary evaporator under reduced pressure at 40 °C to yield crude extracts [40].

### 2.5. Antimicrobial Activity Assay

#### 2.5.1. Strains and Growth of Microorganisms in Solid Culture

The seven extracts were evaluated for antimicrobial activity assay against both yeast strains (Table 1) and bacterial strains (Table 2).

The cultures were performed using the inoculum of the colonies isolated from the strains of interest in standard medium for YPD yeasts (1% yeast extract, 2% peptone, 2% dextrose, and 2% agar) and Luria-Bertani medium (Bio basic inc. S516, Amherst, NY, USA) containing 25 mL of culture medium in each Petri dish. Temperature and growth time ranged from 24 to 48 h and from 30 to 37 °C, depending on experimental planning. Eugenol (Sigma-Aldrich, ST Louis, MO, USA) was used as a positive control for yeasts and kanamycin 50 μg mL^−1^, and the same concentration of ampicillin (AppliChem, Darmstadt, Germany) was used as a positive control for bacteria.

#### 2.5.2. Disc Diffusion Method

The disc diffusion method to assess the antimicrobial susceptibility of microorganisms was performed following the CLSI protocol (M2-A8) [41]. The microorganisms were cultured 24 to 48 h before in LB (bacteria) or YPD (yeast). The inoculum was made by picking 3 colonies and transferred to a test tube containing 5 mL of saline solution (0.9% NaCl). The suspension was adjusted to a cell density similar to the McFarland scale of 0.5 (corresponding to 1.5 × 10^8^ cells per mL). Next, using a sterile swab, the solution was evenly spread onto a plate containing Mueller Hinton agar for bacteria or Mueller Hinton agar (BD 212322) supplemented with 2% D-glucose and 0.5 μL of methylene blue for yeasts. Then, sterile filter paper discs of 5 mm diameter that received 5 to 10 μL of the compounds of interest with a final concentration ranging from 200 μg to 400 μg, a positive control was also placed (disc containing 200 μg/disc of eugenol for yeasts or kanamycin 50 μg/disc for bacteria), and a negative control (disc containing water). The plates were incubated in a bacteriological oven at 30 °C for 24 to 48 h, depending on the microorganism cultivated, after which the halos were measured and the data were duly recorded. The results are presented as the mean of three biological replicates, and statistical analysis (mean comparison) was performed using PrismGraph version 8.0.

#### 2.5.3. Minimum Inhibitory Concentration (MIC)

Microdilution tests were performed according to the CLSI (Clinical and Laboratory Standards Institute) guidelines, specifically OPAS1 M27-A2 for yeasts [42] and OPAS M7-A6 [43] for bacteria, as described in the literature. Briefly, the microorganisms were cultured on solid LB medium and incubated for 24 h at 37 °C. Three fresh colonies were selected and transferred into 5 mL of saline solution (0.9% NaCl), and the cell suspension was adjusted to a final concentration of approximately 1–2 × 10^8^ CFU/mL (0.5 McFarland scale). The extracts were diluted in water to an initial concentration of 0.0007 μg/μL, followed by serial dilution. The extracts were then tested. The growth control contained 100 μL of liquid culture only, while the sterility (negative) control consisted of 100 μL of RPMI medium. The 96-well plates were incubated at 30 °C for 24 h. After incubation, the optical density (OD530) was measured using a plate reader (Epoch 2, Biotek, Santa Clara, CA, USA). After measuring the OD530, 50 μL of 0.005% resazurin solution was added to assess microbial viability, and the plate was incubated again on a shaker at 30 °C for 2 h. The experiments were conducted with two technical replicates and three biological replicates.

### 2.6. Data Acquisition by UHPLC-HRMS/MS and Molecular Networking

Each extract was solubilized in methanol (1 mg/mL), filtered through a 0.45 µm syringe filter Millipore PTFE, 13 mm, 22 µm (Allcrom, São Paulo, Brazil), and analyzed by UHPLC-HRMS/MS. Analysis conditions were in mobile phase gradient mode (A) H_2_O + 0.01% formic acid and (B) methanol, flow rate 300 μL/min: 0–3 min: 2% B; 3–18 min from 2% to 100%; 18–21 min: 100% B constant; 21–25 min returns to the initial condition of 2% B; 25–28 min, with 2% B for column reconditioning, using a reverse phase column (Kinetex EVO C_18_, 100 mm × 2.1 mm, 2.6 μm, 100 Å) at a constant temperature of 50 °C.

The files generated in the UHPLC-HRMS/MS analyses were converted from the original “.d” format to the open “.mzXML” extension for the construction of molecular networking. The newly converted files were checked by SeeMS software 3.0 (Proteowizard^®^). The “.mzXML” files were uploaded to the Global Natural Products Social Molecular Networking (GNPS) platform server using the WinSCP software 6.3.3 [44]. A molecular network was created using the online workflow on the GNPS platform (http://gnps.ucsd.edu) based on the MSCluster algorithm [45]. Data were filtered by removing all ions from the MS/MS fragments within +/− 17 Da of the *m*/*z* precursor. MS/MS spectra acquired on a quadrupole hyphenated time-of-flight (QTof)-type mass analyzer were filtered by selecting only the top six fragment ions in the +/− 50 Da window across the spectrum. The precursor ion mass tolerance was set to 0.02 Da, and the MS/MS fragment ion tolerance to 0.02 Da. A network was created where edges were filtered to have a cosine score above 0.7 and more than six corresponding peaks. In addition, edges between two nodes were kept in the network if and only if each of the nodes appeared in the top five most similar nodes. Finally, the maximum size of a molecular family was set to 100, and the lowest-scoring edges were removed from the molecular families until the molecular family size was below this threshold. The network spectra were then searched against the GNPS spectral libraries, and for dereplication, the library spectra were filtered in the same manner as the input data. All matches maintained between the network spectra and library spectra were required to have a score above 0.7 and at least six matched peaks [44]. Clusters detected in the blank were removed from the network, which included spectral data from solvents used in the extraction and chromatographic procedures (hexane, chloroform, ethyl acetate, and methanol). The molecular networking view and edition were performed in Cytoscape version 3.10.2 [46].

### 2.7. Chromatographic Fractionation and Isolation of Compounds

The extract obtained from *Hypomontagnella barbarensis*—CPFF41 was selected from its profile in molecular networking for further purification. Therefore, part of the extract (150 mg) was subjected to HPLC-DAD in semi-preparative RP-18 in H_2_O with 0.01% formic acid (A) and methanol (MeOH) (B) in gradient mode: 0 min.: 40% B; 7 min.: 15% B; 9 min.: 85% B and gradually returned to the initial condition up to 10 min and oven temperature at 45 °C (flow rates 1.8 mL/min, UV 255 nm) to afford compound **1** (25 mg) and compound **2** (8 mg). These compounds were then analyzed using NMR and HRMS-MS for structural identification.

## 3. Results

### 3.1. Endophytic Fungi Obtained from Leaves

Seven endophytic fungi (designated as CPFF12, CPFF14, CPFF16, CPFF41, CPFF42, CPFF52, and CPFC2) were isolated from *Calea pinnatifida* leaves (Figure S1—Supplementary Material). Five different genera were identified through macro-and microscopic visualization of conidiophores (reproductive structures), hyphae, and growth aspects (Figure S2A–E—Supplementary Material). Furthermore, molecular analyses using sequences obtained from the ITS region and fragment of the *TUB2* gene allowed the identification of the genus *Colletotrichum* for three isolates, CPFF16 being *Colletotrichum siamense* (Figure S1C—Supplementary Material) and CPFF12/CPFF14 as *Colletotrichum karstii* (Figure S1A,B—Supplementary Material), having been confirmed as identical strains only after molecular analysis. Another species found in CPFF41 was *Hypomontagnella barbarensis* (Figure S1D—Supplementary Material), segregated from the *Hypoxylon* group using a polyphasic taxonomic approach [47]. CPFF42 was identified as *Neopestalotiopsis clavispora* (Figure S1E—Supplementary Material), a species inserted in the recently known genus within the *Pestalotiopsis* group. CPFF52 was found to be *Nigrospora sacchari-officinarum* (Figure S1F—Supplementary Material), and finally, CPFC2 was identified as *Annulohypoxylon moriforme* (Figure S1G—Supplementary Material). The database used to identify endophytic fungi was the NCBI platform using the BLAST tool, and the sequences obtained were deposited in GenBank (Table 3). The BLAST results of the nucleotide sequences of endophytic fungi are shown in Table S1—Supplementary Material.

### 3.2. Dereplication Based on Molecular Networking Organization from HRMS-MS Data

The crude EtOAc extracts obtained from the seven endophytic fungi isolated from *C. pinnatifida* were analyzed by UHPLC-HRMS/MS and subsequently dereplicated using the GNPS platform to generate molecular networks and to make annotations of secondary metabolites (Figure S3A,B—Supplementary Material). Dereplication of selected extracts resulted in a total of 5 library matches in positive ionization mode, and annotations made by GNPS suggested three terpenoids: fusaproliferin (cembrane diterpene), alismol (guaiane sesquiterpene) annotated for the *C. karstii* and *N. sacchari-officinarum* and 1-acetoxy-7-isopropylidene-1,4a-dimethyl-6-oxodecahydro-2-naphthalenyl-2,3-dimethyl-2-oxiranecarboxylate (eudesmane sesquiterpene), annotated for the *Colletotrichum* genus (Table 4).

**Figure 1:**
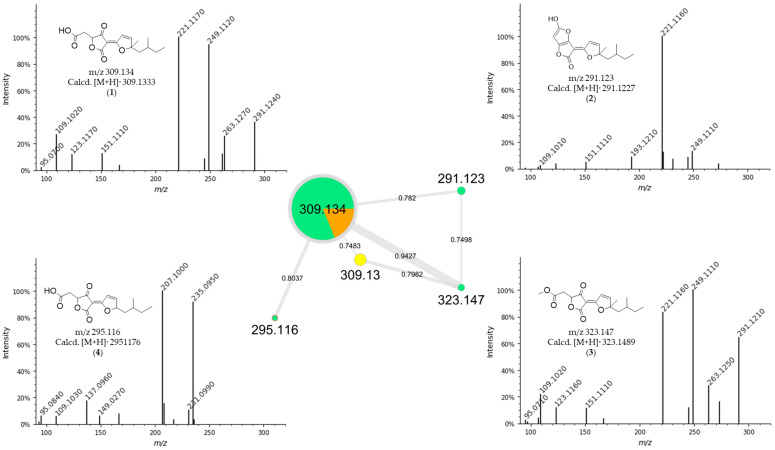
Consensus MS^2^ mass spectra and molecular network created by GNPS. Colors of the nodes: Green: CPFF41; Orange: CPFF42; Yellow: CPFF52. Edge labels between nodes represent *m*/*z* differences. Node labels represent parent masses (*m*/*z*). Edge labels between nodes represent cosine scores. The size of the nodes is proportional to the spectral detection rate.

Furthermore, considering that no known compounds were annotated by GNPS from the extract of *Hypomontagnella barbarensis* (CPFF41), in addition to fatty acid derivatives (Table 4), the extract of this fungus was selected for further analysis. Thus, molecular networking for this extract showed a cluster in which the major compound at *m*/*z* 309.134 was observed, in agreement with the node size in the molecular network, indicating a more significant number of detected spectra (Figure 1). This compound (**1**) was then isolated in a more substantial proportion from the *H. barbarensis* extract and identified as *E*/*Z* nodulisporacid A (see Section 3.3) [48]. In addition, other nodes referring to analogous compounds were observed in the cluster, which includes the methyl ester of nodulisporacid A at *m*/*z* 323.147 ([M+H]^+^) (compound **3**), already described in the literature [48]; a derivative of nodulisporacid A with one less methyl at *m*/*z* 295.116 ([M+H]^+^), reported here for the first time, for which the name *nor*-nodulisporacid A is proposed (compound **4**); and the compound at *m*/*z* 291.123 ([M+H]^+^) whose proposed structure includes an additional furan ring, nodulisfuranol. An additional furan ring is proposed based on a biogenesis hypothesis involving the nucleophilic attachment of a hydroxyl group at C-3 (formed from the enolic tautomer of tetronic acid A) to the C-6 position of the carboxylic acid, followed by dehydration. The last derivative, nodulisfuranol—compound **2**, was isolated from the extract, and its structure was determined by NMR (see Section 3.3) and HR-ESI-MS-MS^2^ (Figure 1).

**Figure 2:**
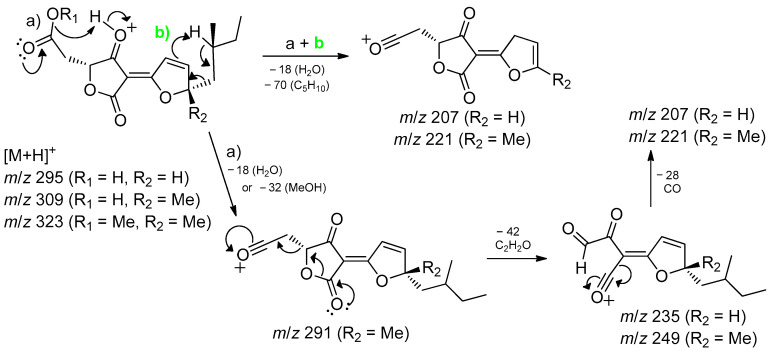
Fragmentation processes for compounds **1** (*m*/*z* 309), **3** (*m*/*z* 323), and **4** (*m*/*z* 295) in ESI— (+)—QTof. Mechanism a - dehydration of the carboxylic acid or elimination of MeOH and mechanism b (green) - McLafferty rearrangement.

**Figure 3:**
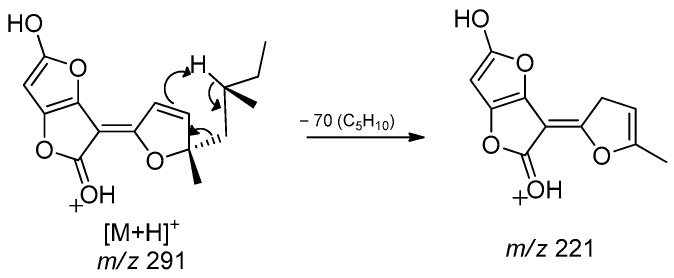
Fragmentation process for compound **2** (*m*/*z* 291) in ESI— (+)—QTof.

Structures **1** to **4** share similar MS^2^ spectra, as observed in the molecular network (Figure 1). Based on this observation, fragmentation proposals were proposed to corroborate and complement their structural similarity and annotation, considering characteristic and complementary fragmentation mechanisms. In the fragmentation spectra of structures **1**, **3**, and **4**, dehydration of the carboxylic acid (**1** and **4**) or elimination of MeOH from the methyl ester (**3**) was observed, resulting in the loss of 18 Da (H_2_O) or 32 Da (MeOH), respectively, corresponding to mechanism (**a**) in Figure 2. Another pathway, which combines mechanisms (**a**) and (**b**), leads to the concurrent loss of H_2_O or MeOH along with the McLafferty rearrangement (loss of 70 Da, C_5_H_10_) in the structures of **1**, **3,** and **4**. This rearrangement occurs due to the deprotonation of the methine carbon C-6’ in the side chain by nucleophilic attack of the *π* electrons of the dihydrofuran ring, resulting in an expected loss of 70 Da in structures **1**, **3**, and **4**. Additionally, based on the proposed alkene loss mechanism (70 Da), it was suggested that structure **4** lacks a methyl group at position C-10’ to maintain similarity with structures **1** and **3**. Consequently, structure **4** has no stereogenic center at position C-4’. Furthermore, via pathway (**a**), the loss of C_2_H_2_O (42 Da) may occur, leading to fragments with *m*/*z* ratios of 249 (compounds **1** and **3**) and *m*/*z* 235 (compound **4**). Subsequently, the intermediate ion can lose 28 Da in the form of CO, supporting an alternative pathway to form ions with ratios of *m*/*z* 221 (compounds **1** and **3**) to *m*/*z* 207 (compound **4**) (Figure 2). Finally, compound **2** exhibits a base peak with an *m*/*z* ratio of 221, corresponding to the previously proposed McLafferty rearrangement mechanism for structures **1**, **3**, and **4**. The additional furan ring also corroborates the less fragmentation pattern in comparison to the structures **1**, **3,** and **4** (Figure 1 and Figure 3).

### 3.3. Identification of Compounds from Fungi Hypomontagnella barbarensis

Considering a molecular family of tetronic acid derivatives and the annotations corresponding to nodulisporacid A and three other derivatives (Figure 1), associated with antimicrobial activity evaluation with lower values of MIC against *S. aureus*, the extract from *H. barbarensis* was then selected for chromatographic fractionation. Thus, purification by HPLC allowed the isolation of compound **1** as a pair of isomers (*E* and *Z*). The compound was subjected to ^1^H and ^13^C NMR and UHPLC-HR-MS/MS analyses for structural elucidation. The ^13^C NMR data indicated the presence of 32 carbon signals, including six methyl groups, six methylenes, four methines, and twelve fully substituted carbons. The carbon resonance at *δ* 170.7/172.6 was attributed to the carboxylic function. The signals were also assigned to an ester (*δ* 163.9/164.0) and an *α,β*-unsaturated ketone (*δ* 196.9/198.3). Other signals included a 1,2-disubstituted olefin unit (*δ* 123.0/123.1 and 164.0/164.1) and fully substituted carbons (*δ* 180.9/181.7, 103.5/104.1, and 94.5/94.9).

**Figure 4:**
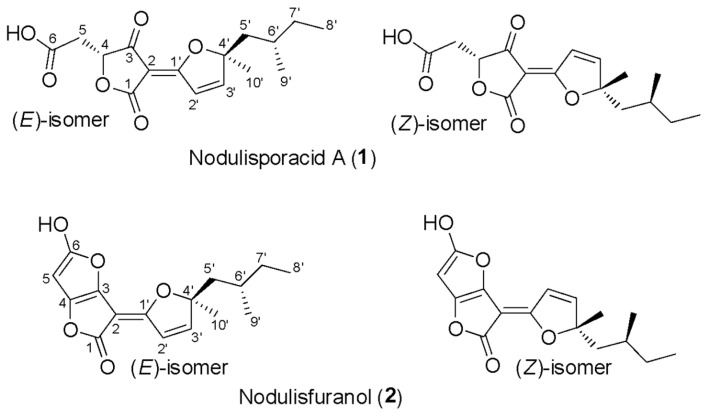
Structures of the *Z-* and *E-* isomers of nodulisporacid A (**1**) and nodulisfuranol (**2**).

Thus, the framework of this compound contains furandione and furylidene fragments connected by a double bond, characteristic of tetronic acid derivatives. Additionally, both the *E-* and *Z-* isomers were observed for the double bond, and the *E-* and *Z*-isomers were interconverted spontaneously and reached an equilibrium state at ca. ratio of 1:1. Therefore, the data were consistent with the structures diastereoisomers *Z* and *E* of nodulisporacid A (Figure 4) and confirmed by comparison of NMR data with those reported in the literature [49]. For compound **2**, ^13^C NMR showed the same skeleton containing sixteen carbon signals duplicated, also revealing spontaneous inter-conversion between *E*- and *Z*-isomers, with a ratio of 1:1. The difference from compound **1** is the absence of a ketone carbonyl signal, indicating that the tetronic ring could be in the enolic form, which may have favored the attack of the enol hydroxyl on the carboxyl leading to the formation of a new furan ring after dehydration in this second ring, evidenced by signals at *δ* 5.67/5.68 (*s*, H-5) referring to the two E/Z stereoisomers in ^1^H-NMR. These signals in the HSQC spectrum present a cross peak with shifts at 98.3/98.4 (C-5). Furthermore, it is possible to observe cross peaks in the HMBC spectrum between H-5 with signals at *δ* 153.2/153.6 (C-3), in addition to the signal at *δ* 182.3/182.5 (C-4). The structure with the additional furan ring was also suggested based on the analysis of the mass spectrum that showed [M+H]^+^ at *m*/*z* 291.1232 corresponding to the molecular formula of C_16_H_18_O_5,_ indicating the presence of eight unsaturations. Thus, the proposed structure is a derivative of compound **1,** and, being the first time reported in the literature, the name nodulisfuranol is proposed (Figure 4).

Compound **1**: Nodulisporacid A (*E:Z* mixture); yellow amorphous solid; *Z*-isomer: ^1^H NMR (MeOH-d_4_, 300 MHz) δ 4.84 (*m*, H-4), 2.87 (*m*, H-5a), 2.73 (*m*, H-5b), 7.32 (*d*, *J* = 5.7 Hz, H-2’), 7.81 (*d*, *J* = 5.7 Hz, H-3’), 2.06 (*m*, H-5’ a), 1.65 (*m*, H-5’ b), 1.25 (*m*, H-6’), 1.25 (*m*, H-7’a), 1.14 (*m*, H-7’b), 0.78 (*t*, *J* = 7.0 Hz, H-8’); 0.85 (*d*, *J* = 6.1 Hz, H-9’); 1.54 (*s*, H-10’). ^13^C NMR (MeOH-d_4_, 75 MHz) δ 172.5 (C, C-1), 94.9 (C, C-2), 198.3 (C, C-3), 80.7 (CH, C-4), 36.8 (CH_2_, C-5), 173.1 (C, C-6), 181.6 (C, C-1’), 123.1 (CH, C-2’), 164.1 (CH, C-3’), 103.4 (C, C-4’), 45.2 (CH_2_, C-5’), 31.3 (CH_3_, C-6’), 31.5 (CH_2_, C-7’), 11.4 (CH_3_, C-8’); 21.3 (CH_3_, C-9’), 23.8 CH_3_, C-10’). *E*-isomer: ^1^H NMR (MeOH-d_4_, 300 MHz) δ 4.87 (*m*, H-4), 2.93 (*m*, H-5a), 2.79 (*m*, H-5b), 7.47 (*d*, *J* = 5.8 Hz, H-2’), 7.83 (*d*, *J* = 5.7 Hz, H-3’), 2.12 (*m*, H-5’a), 1.72 (*m*, H-5’b), 1.25 (*m*, H-6’), 1.25 (*m*, H-7’a), 1.14 (*m*, H-7’b), 0.77 (*t*, *J* = 7.0 Hz, H-8’); 0.80 (*d*, *J* = 6.1 Hz, H-9’); 1.54 (*s*, H-10’). ^13^C NMR (MeOH-d_4_, 75 MHz) δ 170.7 (C, C-1), 94.5 (C, C-2), 196.8 (C, C-3), 80.3 (CH, C-4), 36.7 (CH_2_, C-5), 172.9 (C, C-6), 180.9 (C, C-1’), 123.0 (CH, C-2’), 163.9 (CH, C-3’), 103.4 (C, C-4’), 45.1 (CH_2_, C-5’), 31.3 (CH, C-6’), 31.4 (CH_2_, C-7’), 11.4 (CH_3_, C8’), 21.2 (CH_3_, C-9’), 23.7 (CH_3_, C-10’).

Compound **2**: Nodulisfuranol (*E*:*Z* mixture); yellow amorphous solid; *Z*-isomer: ^1^H NMR (MeOH-d_4_, 300 MHz) δ 5.67 (*s*, H-5), 7.25 (*d*, *J* = 5.8 Hz, H-2’), 7.86 (*d*, *J* = 5.8 Hz, H-3’), 2.01 (*m*, H-5’a), 1.60 (*m*, H-5’b), 1.15–1.19 (*m*, H-6’), 1.15–1.19 (*m*, H-7’a), 1.04 (*m*, H-7’b), 0.74 (*t*, *J* = 7.0 Hz, H-8’), 0.78 (*d*, *J* = 6.0 Hz, H-9’), 1.47 (*s*, H-10’), 7.87 (*s*, OH). ^13^C NMR (MeOH-d_4_, 75 MHz) δ 166.1 (C, C-1), 92.5 (C, C-2), 153.2 (C, C-3), 182.3 (C, C-4), 98.3 (C, C-5), 165.6 (C, C-6), 182.4 (C, C-1’), 123.1 (CH, C-2’), 163.6 (CH, C-3’), 104.7 (C, C-4’), 44.9 (CH_2_, C-5’), 31.3 (CH, C-6’), 31.5 (CH_2_, C-7’), 11.4 (CH_3_, C-8’), 21.1 (CH_3_, C-9’), 23.4 (CH_3_, C-10’). *E*-isomer: ^1^H NMR (MeOH-d_4_, 300 MHz) δ 5.68 (*s*, H-5), 7.40 (*d*, *J* = 5.7 Hz, H-2’), 7.89 (*d*, *J* = 5.7 Hz, H-3’), 2.03 (*m*, H-5’a), 1.68 (*m*, H-5’b), 1.15–1.19 (*m*, H-6’), 1.15–1.19 (*m*, H-7’a), 1.04 (*m*, H-7’b), 0.74 (*t*, *J* = 7.0 Hz, H-8’), 0.79 (*d*, *J* = 6.0 Hz, H-9’), 1.50 (*s*, H-10’), 7.90 (*s*, OH). ^13^C NMR (MeOH-d_4_, 75 MHz) δ 167.9 (C, C-1), 93.0 (C, C-2), 153.6 (C, C-3), 182.5 (CH, C-4), 98.4 (C, C-5), 165.9 (C, C-6),182.5 (C, C-1’), 123.2 (CH, C-2’), 163.9 (CH, C-3’), 105.4 (C, C-4’), 45.0 (CH_2_, C-5’), 31.4 (CH_3_, C-6’), 31.5 (CH_2_, C-7’), 11.4 (CH_3_, C-8’), 21.2 (CH_3_, C-9’), 23.5 (CH_3_, C-10’).

### 3.4. Antimicrobial Activity

**Figure 5:**
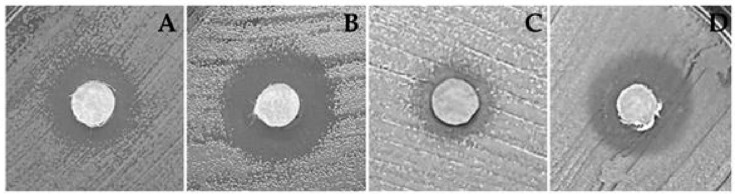
Illustration of the growth inhibition zone method used to evaluate the antimicrobial activity of the crude extracts (CPFF12 and CPFF14) using the disc diffusion test. Inhibition halos formed by the crude extract CPFF12 against (**A**) *Candida albicans*; (**B**) *Saccharomyces cerevisiae*; crude extract CPFF12 against (**C**) *Staphylococcus aureus*; and crude extract CPFF41 against (**D**) *Staphylococcus aureus*.

Initially, disk diffusion assays were conducted with the yeasts and bacterial strains listed in Table 1 and Table 2 in order to screen fungal extracts for antimicrobial activity. The CPFF14 and CPFF41 extracts inhibited the *S. aureus* strain, and the CPFF12 extract proved promising against two yeast strains, *S. cerevisiae* and *C. albicans,* in the disk diffusion assay. The other extracts evaluated did not show significant inhibition values (Figure 5 and Table 5).

In sequence, the species listed in Table 1 and Table 2 were tested to determine the minimum inhibitory concentration (MIC). The results showed that yeasts were more sensitive to the extract CPFF12 (*C. karstii*), while the bacteria *S. aureus* was more sensitive to the extracts CPFF14 and CPFF41. The *H. barbarensis* (CPFF41) extract resulted in 90% growth inhibition at 0.05 mg/mL (Table 6). The other extracts also showed no significant activity against microbial strains.

## 4. Discussion

Asteraceae species, including *C. pinnatifida*, are common or cosmopolitan endophytic species [50]. The genera found, *Colletotrichum*, *Nigrospora,* and *Pestalotiopsis*, according to Caruso [11] and Nicoletti [51], were isolated from most plant species in this family, while the genus *Xylaria* was observed in a lower percentage. In addition, these microorganisms have been described as phytopathogens that can affect a wide range of economically important crops, including fruits, vegetables, ornamental plants, and grasses; some species in this group have also become the object of study for biotechnological applications, seeking new bioactive compounds that can be used in new environments [52].

From *C. pinnatifida*, two species from the *Colletotrichum* genus were isolated, including *C. siamense* and *C. karstii*, besides species of *Hypomontagnella barbarensis*, *Neopestalotiopsis clavispora*, *Nigrospora sacchari-officinarum,* and *Annulohypoxylon moriforme*. These genera of endophytes have already been described as endophytes of other species and produce metabolites of interest, such as *Colletotrichum gloeosporioides*, which has stood out in studies on the production of toxic metabolites and promising biological activity [53]. Wang [54] reported that pestalachlorides E and F, isolated from fungi of the genus *Pestalotiopsis*, showed potent agronomic activity, which was also reported by Yang [55] regarding the isolation of the anticancer Taxol present in the species *Pestalotiopsis microspora*. Other compounds with interesting activities come from the *Nigrospora* genus, ranging from herbicides to insecticides and antimicrobials [56,57,58,59]. These secondary metabolites have demonstrated a wide range of biological activities, including antifungal, antibacterial, antiviral, antitumor, and cytotoxic properties [60,61,62]. Hypoxylaceae family was recently elevated from Xylariaceae, representing one of the predominant groups of fungal endophytes.

Furthermore, from this family, the species *Hypomontagnella barbarensis*, a new genus of the Hypoxylaceae family, segregated from *Hypoxylon* by a polyphasic taxonomic approach, was identified [47]. Regarding the chemical composition of the extracts, some metabolites, already described in the literature, isolated from other organisms were annotated by GNPS, such as fusaproliferin, a compound with anti-inflammatory potential, isolated from *Fusarium proliferatum* [63] (Figure S3A Supplementary Material and Table 4). Most of these bioactive compounds are derived from the secondary metabolism of these organisms, through which they generate compounds belonging to the most diverse chemical classes, such as terpenes, alkaloids, polyketides, and peptides [64]. Molecular networking analysis also allowed the dereplication of a molecular family of tetronic acid derivatives. made it possible to annotate derivatives from *H. barbariensis*, which drives chromatographic fractionation. Furthermore, compound **1**, isolated from *H. barbariensis*, identified as two isomers (*E* and *Z*) of nodulisporacid A, has been characterized by Kasettrathat et al. [65], and it has already been reported by Sumiya et al. [49] being isolated from the marine microorganism *Nodulisporium* sp. and by Huang et al. [48] isolated from an endophytic fungus *Hypomontagnella monticulosa*, belonging to the same genus as the fungus found. This compound has been tested for its bioactivity and cytotoxicity, showing moderate activity against cancer cell lines, as developed by Kasettrathat et al. [65], and moderate antimalarial activity, as developed by Huang et al. [48]. However, compound **2,** which was isolated from the same extract, was identified as a derivative of nodulisporacid A named nodulisfuranol and has not yet been described in the literature, suggesting that it is a new compound, as well as compound **4** (*nor*-nodulisporacid A) of *m*/*z* 295 annotated in the nodulisporacid A cluster.

The antimicrobial activity of the endophytic fungal extracts was evaluated by disk diffusion and MIC assays against eight bacterial strains, including *S. aureus* and 10 yeast strains. However, only the extracts of *C. karstii* and *H. barbarensis* were active against at least one of the strains, whereas the extract of *H. barbarensis* was active against *S. aureus*. It was also demonstrated that this extract is composed of Noduliporacid A and the derivative, named nodulisfuranol. Considering that nodulisporacid A has already been evaluated against *S. aureus* in the work of Huang et al. [48] and that it showed no activity, it can be suggested that the activity observed for the CPFF41 extract may be due to the presence of the derivative, or even due to the synergistic action between the two compounds.

Furthermore, combining the data from the GNPS annotations and microbiological assays, we suggest that the presence of the annotated compounds, such as fusaproliferin and alismol, is related to the antimicrobial activity of the extracts. For the CPFF12 extract, the annotation of these two compounds was observed, which has already been described as active and with cytotoxic/antimicrobial action [66,67]. This may explain the inhibition, especially of *C. albicans*, with a low extract concentration. This clarifies that synergy between compounds can occur; thus, the activity can be associated with more than one compound.

Due to these promising activities, accessing the metabolic profile of these individuals is of great interest. The presence of compounds already described as active compounds and the possibility of isolating new compounds open the opportunity for new discoveries. Additionally, considering the large number of secondary metabolites that fungi might contain, dereplication tools, such as LC-MSMS and molecular networking, have become important for prospecting metabolites, saving time, and working on the isolation and characterization of natural products. However, whereas genome analyses have revealed a great diversity of biosynthetic gene clusters (BGCs) that far exceed the number of known substances, most of its secondary metabolites remain unknown [68]. Thus, dereplication of the extracts from fungi, with the aid of GNPS to annotate metabolites, can be complemented with genomic analysis integrated with metabolomics to identify gene clusters related to the biosynthesis of the annotated molecules and thus can allow access to the full biosynthetic potential of the fungi.

## 5. Conclusions

Investigating endophytic fungi offers a promising avenue for the discovery of new bioactive compounds with potential pharmaceutical applications. Through the methods outlined in this study, we isolated six endophytic fungi from *C. pinnatifida* leaves, belonging to five distinct genera: *Colletotrichum*, *Neopestalotiopsis*, *Nigrospora*, *Hypomontagnella,* and *Annulohypoxylon*. Although these genera are recognized for their phytopathogenic roles, they have also demonstrated biotechnological promise for the production of bioactive metabolites. Additionally, our findings regarding the chemical composition of extracts from fungi indicate that the molecular network approach based on GNPS showed to be a rapid and efficient tool to organize into clusters and annotate bioactive metabolites from a crude extract, not only based on the annotation of known molecules but also for new ones, such as acyl-tetronic acid derivatives suggested for the first time in the present study. Moreover, two pairs of diastereoisomeric tetronic acid derivatives were isolated and identified by NMR, including two new compounds from the most active extract against *S. aureus*. Thus, this approach can be used for drug discovery. Remarkably, the array of bioactive compounds synthesized by these endophytic fungi underscores their potential as valuable reservoirs for new pharmaceuticals, agrochemicals, and industrial compounds. Overall, this study underscores the significance of investigating endophytic fungi associated with medicinal plants like *C. pinnatifida*. It provides fertile ground for the discovery of new bioactive compounds with a wide range of pharmacological properties.

## Figures and Tables

**Table 1:** Yeast strains used in yeast inhibitory activity tests.

Species	Cell Line
*Cryptococcus neoformans*	KN99α (serotype A)
*Cryptococcus neoformans*	JEC21 (serotype D)
*Cryptococcus gattii*	NIH312 (serotype C)
*Cryptococcus gattii*	R265 (serotype B)
*Saccharomyces cerevisiae*	BY4647
*Candida krusei*	Clinical isolate 9602
*Candida parapsilosis*	Clinical isolate 68
*Candida albicans*	CBmay 560
*Candida tropicalis*	ATCC 1303
*Candida dubliniensis*	ATCC 7876

**Table 2:** Bacterial strains used for disc diffusion and minimum inhibitory concentration assays.

Species	Cell Line
*Enterococcus faecium*	ATCC CCB076
*Pseudomonas aeruginosa*	ATCC 27853
*Klebsiella pneumoniae*	ATCC 700603
*Escherichia coli*	ATCC 25922
*Shigella flexneri*	ATCC 12022
*Salmonella enterica*	ATCC 14028
*Staphylococcus aureus*	ATCC 25923
*Acinetobacter baumanii*	ATCC 19606

**Table 3:** Species identification of isolates based on sequences obtained from ITS region and fragment of the *TUB2* gene.

Strain Number	Species	% Identity *			
		ITS	Cod.	*TUB2*	Cod.
CPFF12	*Colletotrichum karstii*	100	PP831945	100	PP840075
CPFF14	*Colletotrichum karstii*	99.39	PP829193	99.72	PP840076
CPFF16	*Colletotrichum siamense*	100	PP829196	99.60	PP840077
CPFF41	*Hypomontagnella barbarensis*	99.23	PP829195	93.24	PP840078
CPFF42	*Neopestalotiopsis clavispora*	100	PP829194	99.09	PP942538
CPFF52	*Nigrospora sacchari-officinarum*	99.08	PP832015	100.00	PP840079
CPFC2	*Annulohypoxylon moriforme*	98.50	PP834406	98.09	PP840080

* BLAST NCBI.

**Table 4:** Annotation of compounds in the GNPS spectral library.

	Compound Name	Molecular Formula	Calculated Mass	Precursor Ion	Error (ppm)	Sample	Structural Formula
**1**	1-Acetoxy-7-isopropylidene-1,4a-dimethyl-6-oxodecahydro-2-naphthalenyl 2,3-dimethyl-2-oxiranecarboxylate	C_22_H_32_O_6_	415.2097 [M+Na]^+^	415.2097	0.1	CPFF12 CPFF16	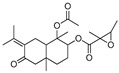
**2**	13-keto-9*Z*,11*E*-octadecadienoic acid	C_18_H_30_O_3_	295.2273 [M+H]^+^	295.2275	0.7	CPFF41 CPFF42	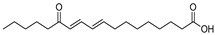
**3**	Alismol	C_15_H_24_O	203.1799 [M+H]^+^	203.1797	1.0	CPFF14 CPFF52	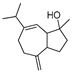
**4**	Phytosphingosine	C_18_H_39_NO_3_	318.3008 [M+H]^+^	318.3000	2.6	CPFF12 CPFF16 CPFF52	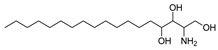
**5**	Fusaproliferin	C_27_H_40_O_5_	445.2954 [M+H]^+^	445.2949	1,1	CPFF12 CPFF14	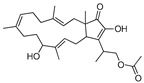

**Table 5:** Disk diffusion method (DDM)—zone of inhibition (diameter in mm).

Extract (400 μg)	Species (Lineage)		
	*S. cerevisiae * (BY4647)	*C. albicans * (CBmay 560)	*S. aureus * (ATCC 25923)
CPFF12	1.4 ± 0.26	1.23 ± 0.25	n/a
CPFF14	n/a	n/a	0.73 ± 0.23
CPFF41	n/a	n/a	1.37 ± 0.32
Control *	2.07 ± 0.12 ^a^	2.07 ± 0.12 ^b^	2.5 ± 0.0 ^b^

* ^a^ Eugenol (200 μg); ^b^ Kanamicin (50 μg). n/a—not active.

**Table 6:** Minimum inhibitory concentration (*MIC*).

Specie	Extract—MIC90 (mg/mL)			Control *
	CPFF12	CPFF14	CPFF41	
*S. aureus* (ATCC 25923)	n/a	0.20 (91.6 ± 1.5)	0.05 (92.0 ± 1.0)	0.004 ^a^
*C. albicans* (CBmay 560)	0.01 (92.0 ± 1.9)	n/a	n/a	0.025 ^b^
*S. cerevisiae* (BY4647)	0.02 (90.3 ± 1.5)	n/a	n/a	0.013 ^c^

* ^a^ Vancomycin; ^b^ Chloramphenicol; ^c^ Fluconazole. n/a—not active.

## Data Availability

All Mass Spectrometry data used in this work are available at the MassIVE repository MSV000095564 according to the DOI-code 10.25345/C5BC3T821. This repository is accessible online (ftp://massive.ucsd.edu/v08/MSV000095564/, accessed on 11 March 2024).

## Supplementary Materials

The following supporting information is available online and can be downloaded at https://www.mdpi.com/article/10.3390/jof11010022/s1. Figure S1: BLAST of the nucleotide sequence of endophytic fungi from *Calea pinnatifida*. Figure S2: Micromorphology slides of endophytic fungi. Figure S3: Molecular network of the crude extracts of endophytic fungi isolated from *Calea pinnatifida*. Figure S4: NMR spectra of the isolated compounds. Table S1: Blast of the nucleotide sequence of endophytic fungi from *Calea pinnatifida.*
