# Supplementary material for: Endophytic Fungi of Calea pinnatifida (Asteraceae): Dereplication of Crude Extracts, Antimicrobial Properties, and Identification of New Tetronic Acid Derivative Produced by Hypomontagnella barbarensis
Source: J Fungi (Basel). 2024 Dec 31;11(1):22. doi: 10.3390/jof11010022 (PMC11766799; doi:10.3390/jof11010022)
Supplement: Supplementary file 1 [file jof-11-00022-s001.zip › jof-3304846-supplementary.pdf]

Endophytic Fungi of *Calea pinnatifida* (Asteraceae): Dereplication of Crude Extracts, Antimicrobial Properties, and Identification of New Tetronic Acid Derivative Produced by *Hypomontagnella barbarensis*

Bianca Barna[1], Lhaís Araújo Caldas[1], Jackson Monteiro[1], Augusto Leonardo dos Santos[1], Renata Castiglioni Pascon[1], Marcelo Afonso Vallim[1], Marcelo José Pena Ferreira[2], Sarah Gonçalves Tavares[3], Glaucia Santos Gonçalves[3], Anderson Messias Rodrigues[4], Jamile Ambrósio de Carvalho[4], Suzan Pantaroto de Vasconcellos[1], Patricia Sartorelli[1*]

[1] Institute of Environmental, Chemical and Pharmaceutical Sciences, Federal University of São Paulo, Diadema 09972-270, SP, Brazil
[2] Department of Botany, Institute of Biosciences, University of São Paulo, São Paulo 05508-090, SP, Brazil
[3] Department of Pathology, Federal University of Espírito Santo, Goiabeiras, Vitória 29075-910, ES, Brazil
[4] Department of Microbiology, Immunology and Parasitology, Federal University of São Paulo, São Paulo 04023-062, SP, Brazil
* Correspondence: psartorelli@unifesp.br

Supplementary Material

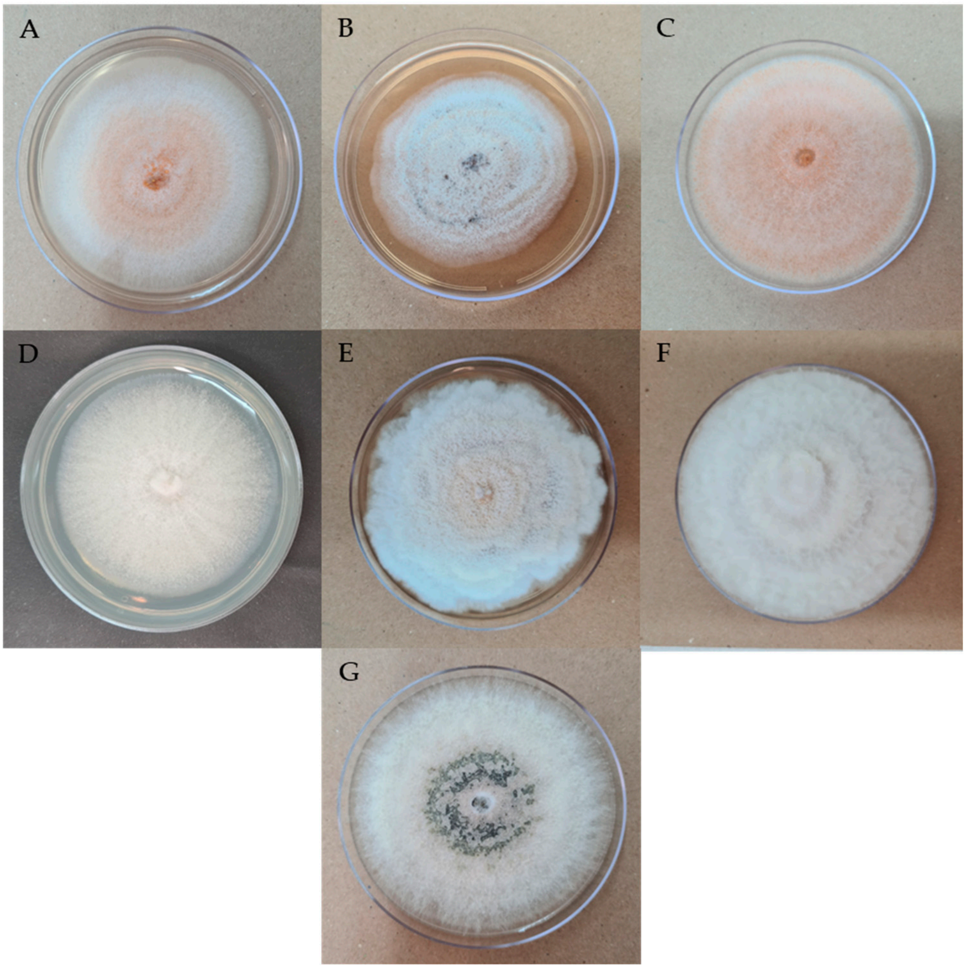

Figure S1. Endophytic fungi from *C. pinnatifida* grown in PDA culture medium for 7 days. A and B: *Colletotrichum karstii* (CPFF12, CPFF14); C: *Colletotrichum siamense* CPFF16; D: *Hypomontagnella barbarensis* (CPFF41); E: *Neopestalotiopsis clavispora* (CPFF42); F: *Nigrospora sacchari-officinarum* (CPFF52) and G: *Annulohypoxylon moriforme* (CPFC2).

Figure S2*: Micromorphology slides of endophytic fungi*

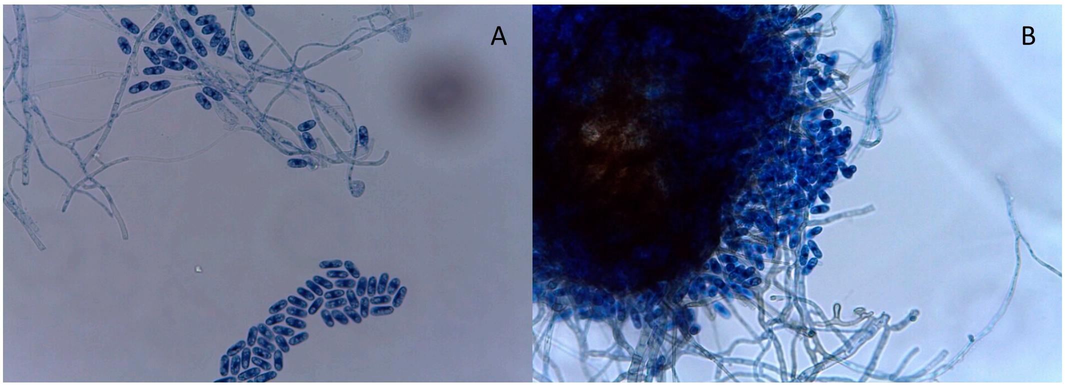

Figure S2A. A and B) Conidia and hyphae highlighted in blue - *Colletotrichum karstii* (CPFF12).

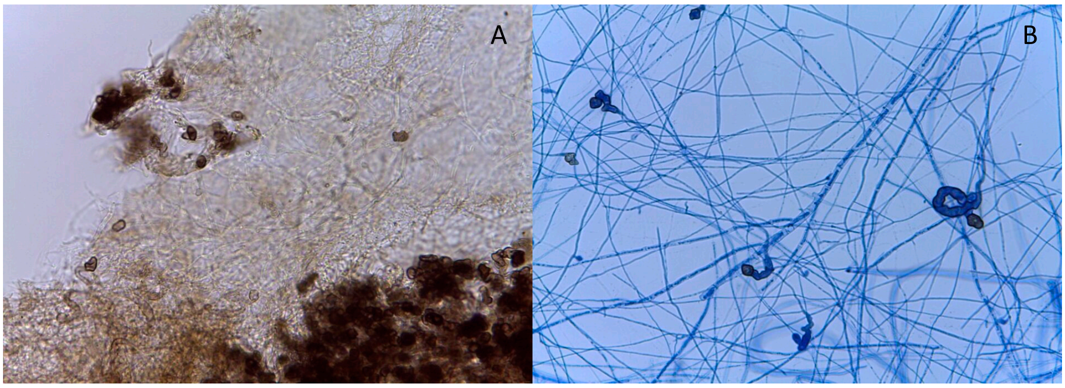

Figure S2B. A) Conidia in dark brown; B) hyphae highlighted in blue - *Colletotrichum karstii* (CPFF14).

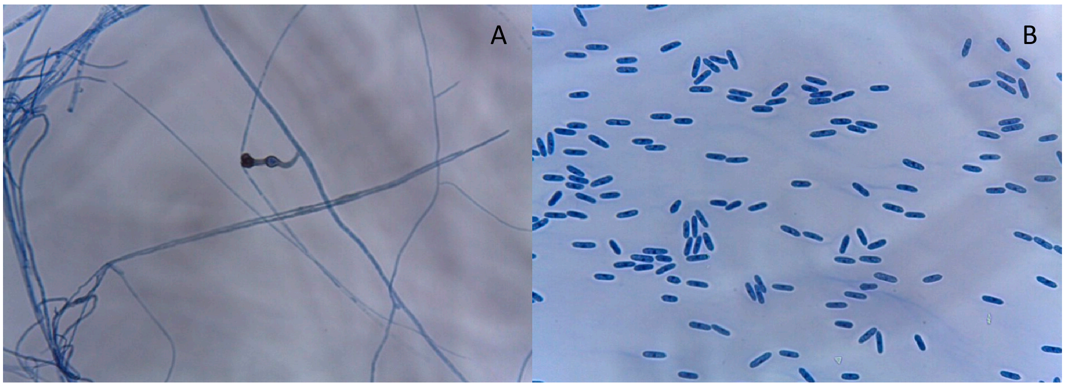

Figure S2C. A) Hyphae highlighted in blue; B) Conidia highlighted in blue - *Colletotrichum siamense* (CPFF16).

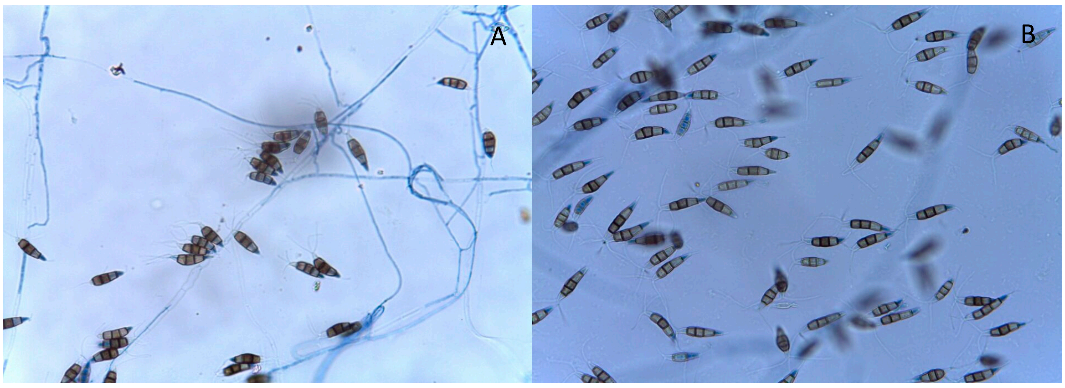

Figure S2D. A) Light brown conidia clustered together; B) Loose light brown conidia with a very distinctive and classic shape for this genus - *Neopestalotiopsis clavispora* (CPFF42).

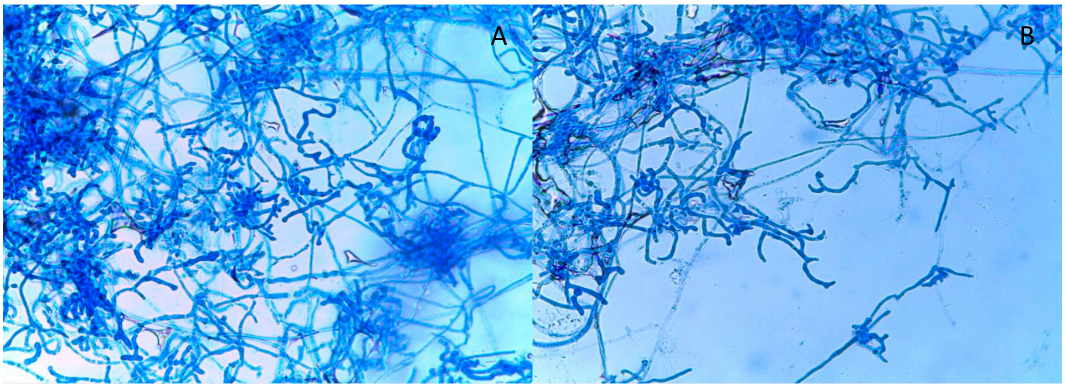

Figure S2E. A and B) Mycelium of filamentous fungus, intertwined hyphae highlighted in blue - *Nigrospora sacchari-officinarum* (CPFF52).

Table S1: Blast of the nucleotide sequence of endophytic fungi from *Calea pinnatifida*.

Endophytic fungi		*Concessional sequence*
Colletotrichum karstii (CPFF12)	**ITS**	GTCATTTAGAGGAAGTAAAAGTCGTAACAAGGTCTCCGTT<br>GGTGAACCAGCGGAGGGATCATTACTGAGTTACCGCTCTA<br>TAACCCTTTGTGAACATACCTACAACTGTTGCTTCGGCGGG<br>TAGGCCGTCCCCTGAAAAGGACGCCTCCCGGCCCGGACC<br>GGACCCCCCGCGGGACCGGACCCGGCGCCCGCCGGAGGA<br>TAACCAAACTCTATTGT
	**TUB2**	TGACCGCTAATCTTCTGAATAGCTACAACGGCACTTCCGAG<br>CTCCAGCTCGAGCGCATGAGCGTCTACTTCAACGAAGTTTG<br>TTATCCTAGAAACCCCAAGTGTTCAAGAAAAACATATTGAC<br>GAATACTGACCTTCGCTCCTTCGCAGGCCTCCGGCAACAAG<br>TACGTGCCCCGTGCTGTTCTCGTCGACTTGGAGCCCGGTAC<br>CATGGACGCCGTCCGCGCCGGTCCTTTCGGTCAGCTTTTCC<br>GTCCCGACAACTTCGTTTTCGGTCAGTCCGGTGCCGGCAAC<br>AACTGGGCCAAGGGTCACTACACTGA
Colletotrichum karstii (CPFF14)	**ITS**	AGAGGTGGGCAACTACCACTCAGAGTCGAAAGTTATCCAA<br>ACTCGGTCATTTAGAGGAAGTAAAAGTCGTAACAAGGTCTC<br>CGTTGGTGAACCAGCGGAGGGATCATTACTGAGTTACCGCT<br>CTATAACCCTTTGTGAACATACCTACAACTGTTGCTTCGGCG

		GGTAGGCCGTCCCCTGAAAAGGACGCCTCCCGGCCCGGAC<br>CGGACCCCCCGCGGGACCGGACCCGGCGCCCGCCGGAGGA<br>TAACCAAACTCTATTGTAACGACGTTTCTTCTGAGTGGCATA<br>AGCAAAATAATCAAAACTTTTAACAACGGATCTCTTGGTTCT<br>GGCATCGATGAAGAACGCAGCGAAATGCGATAAGTAATGTG<br>AATTGCAGAATTCAGTGAATCATCGAATCTTTGAAACGCAC<br>ATTGCGCCCGCCAGCATTCTGGCGGGCATGCCTGTTCGAGC<br>GTCATTTCAAACCCTCAAGCTCTGCTTGGTGTTGGGGCTCTA
	**TUB2**	ATCGCTACCTCTTCCATGGATGGAAATATTGACGATGGAACA<br>GGCAAAACATCTCTGGCGAGCACGGCCTCGACAGCAATGG<br>CGTGTATGTGGTCGACTCCCCATCTGGCCACGTCGGGACTTG<br>ACCGCTAATCTTCTGAATAGCTACAACGGCACTTCCGAGCTC<br>CAGCTCGAGCGCATGAGCGTCTACTTCAACGAAGTTTGTTAT<br>CCTAGAAACCCCAAGTGTTCAAGAAAACCATATTGACGAAT<br>ACTGACCTTCGCTCCTTCGCAGGCCTCCGGCAACAAGTACG<br>TGCCCCGTGCTGTTCTCGTCGACTTGGAGCCCGGTACCATGG<br>ACGCCGTCCGCGCCGGTCC
Colletotrichum siamense (CPFF16)	**ITS**	GTCGCTACTACCGATTGAATGGCTCAGTGAGGCTTTCGGACT<br>GATCTAGGGCGGGGTTCATTCGCCGCCTCTGGTCGGAAAGTT<br>ATCCAAACTCGGTCATTTAGAGGAAGTAAAAGTCGTAACAAG<br>GTCTCCGTTGGTGAACCAGCGGAGGGATCATTACTGAGTTTAC<br>GCTCTACAACCCTTTGTGAACATACCTATAACTGTTGCTTCGGC<br>GGGTAGGGTCTCCGTGACCCTCCCGGCCTCCCGCCCCCGGGC<br>GGGTCGGCGCCCGCCGGAGGATAACCAAACTCTGATTTAACG<br>ACGTTTCTTCTGAGTGGTACAAGCAAATAATCAAAACTTTTAA<br>CAACGGATCTCTTGGTTCTGGCATCGATGAAGAACGCAGCGA<br>AATGCGATAAGTAATGTGAATTGCAGAATTCAGTGAATCATCG<br>AATCTTTGAACGCACATTGCGCCCGCCAGCATTCTGGCGGGCA<br>TGCCTGTTCGAGCGTCATTTCAACCCTCAAGCTCTGCTTGGTGT<br>TGGGGCCCTACAGCTGATGTAGGCCCTCAAAGGTAGTGGCGGA<br>CCCTC
	**TUB2**	ACAGCAATGGAGTGTATGTCATGCCCATTATCTGGCCACATTGG<br>TGGTTGACCGCTAAACTCGAACAGCTACAACGGCACCTCTGAG<br>CTCCAGCTCGAGCGCATGAGCGTCTACTTCAACGAAGTTTGTTA<br>CCTTATAGCCCCCAGAGTGCAAGATAAACATATTGACGAGTACT<br>GACCTTCGCTCCTACCCAGGCTTCCGGCAACAAGTACGTGCCC<br>CGTGCCGTCCTCGTCGATTTGGAGCCCGGTACCA
Hypomontagnella barbarensis (CPFF41)	**ITS**	TAGAGGAGTCGGCAACGACACCTCAGGGCCGGAAAGTTATCCA<br>AACTCGGTCATTTAGAGGAAGTAAAAGTCGTAACAAGGTCTCC<br>GTTGGTGAACCAGCGGAGGGATCATTACTGAGTTATCCAAAACT<br>CCCAACCCTTTGTGAACTTACCACTGTTGCCTCGGCGAGCTGTG<br>CTACCCTATAGCTACCCTATAGCTACCCGGGAGCACGCTATAAG<br>CTCGCCAGAGGACCACTAAACTCTGTTTTTATTTTGTATCTCTGA<br>ATCGTATAACTAAATCAGTTAAAACTTTCAACAACGGATCTCTTG<br>GTTCTGGCATCGATGAAGAACGCAGCGAAATGCGATAAGTAATG<br>TGAATTGCAGAATTCAGTGAATCATCGAATCTTTGAACGCACATT<br>GCGCCCATTAGTATTCTAGTGGGCATGCCTATTCGAGCGTCATTTC<br>AACCCTTAAGCCTCAGTTGCTTAGCGTTGGGATTCTACGGGTTAC<br>AGCGTAGTTCCCTAAAAGTAGTGGCGGAGTTGGTGCTCACTCTC<br>AGCGTAGTAATCTCTTCTCGCTTTTGTAGTGGCGCTGGCGGCCG
	**TUB2**	TATCAATGGTCAACTCAGAGCTCCCAAAATATCAACTGACCAAT

		AAACAGCTACAACGGTACCTCTGAGCTTCAGCTCGAGCGCATG<br>AGCGTCTACTTCAACGAGGTATGTAGCTTCAGAAATCCAGTATG<br>GATATGCAAGATCGGCTACTAATCATCCCTGATATCTACAGGGTT<br>CTGGCAACAAGTATGTTCCTCGCGCCGTTCTCGTCGATCTCGAG<br>CCTGGCACCATGGATGCCGTCCGTGCTGGTCCTTTCGGCCAGCT<br>TTTCCGACCTGACAAC
Neopestalotiopsis clavispora (CPFF42)	**ITS**	TCGGCAACGACCACCCAGGGCCGGAAAGTTATCCAAACTCGGT<br>CATTTAGAGGAAGTAAAAGTCGTAACAAGGTCTCCGTTGGTGAA<br>CCAGCGGAGGGATCATTATAGAGTTTTCTAAACTCCCAACCCAT<br>GTGAACTTACCTTTTGTTGCCTCGGCAGAAGTTATAGGTCTTCTTA<br>TAGCTGCTGCCGGTGGACCATTAAACTCTTGTTATTTTATGTAATC<br>TGAGCGTCTTATTTTAATAAGTCAAAACTTTCAACAACGGATCTC<br>TTGGTTCTGGCATCGATGAAGAACGCAGCGAAATGCGATAAGTA<br>ATGTGAATTGCAGAATTCAGTGAATCATCGAATCTTTGAACGCAC<br>ATTGCGCCCATTAGTATTCTAGTGGGCATGCCTGTTCGAGCGTCAT<br>TTCAACCCTTAAGCCTAGCTTAGTGTTGGGAATCTACTTCTCTTAG<br>GAGTTGTAGTTCCTGAAATACAACGGCGGATTTGTAGTATCCTCT<br>GAGCGTAGTA
	**TUB2**	CGAGCTCCAGCTCGAGCGTTGAGCGTCTACTTCAACGAGGCTTC<br>CGGCAACAAGTACGTTCCTCGTGCCGTCCTCGTCGATCTCGAGC<br>CCGGTACCATGGATGCCGTCC
Nigrospora sacchari-officinarum (CPFF52)	**ITS**	GCGGAGGGATCATTACAGAAGTTATCCAACTCCCAAACCCATGT<br>GGAACTTATCTCTTTGTTGCCTCGGCGCAAGCTACCCGGGACCC<br>AGCGCCCCGGGCGGCCCGCCGGCGGACAAACCAAACTCTTGTT<br>ATCTTAGTTGATTATCTGAGCGTCTTATTTAATAAGTCAAAACTTT<br>CAACAACGGATCTCTTGGTTCTGGCTCGATGAAGAACGCAGCG<br>AAATGCGATAAGTAATGTGAATTGCAGAATTCAGTGAATCATCG<br>AATCTTTGAACGCACATTGCGCCCATTAGTATTCTAGTGGGCAT<br>GCCTGTTCGAGCGTCA
	**TUB2**	TTTCCCGCCTACCCATTTCCCACCCTCCTCCTTGCCCTCCACACC<br>ACCGCGTGACGGCGGGGTGGATGAAAAACAAGAGACAATGCT<br>ATGGGAAGAGAGAGAGCCGTCCACTGACAACGGCCTTTCTACA<br>GGTACAACGGCACCTCCGAGCTCCAGCTCGAGCGCATGAGCGT<br>TTACTTCAACGAGGCCTCGGGCAACAAGTACGTTCCCCGTGCCG<br>TCCTCGTCGATCTCGAGCCCGGTACCATGGATGCCGTCCGTGCC<br>GGTCCTTTCGGCCAGCTGTTCCGTCCCGACAACTTCGTTTTCGGC<br>CAGTCTGGTGCTGGCAACAACTGGGCCAAGGGTCACTACACTG
Annulohypoxylon moriforme (CPFC2)	**ITS**	GTTATCCAAACTCGGTCATTTAGAGGAAGTAAAAGTCGTAACAA<br>GGTCTCCGTTGGTGAACCAGCGGAGGGATCATTACTGAGTTATT<br>ACAACTCCAACCCTTTGTGAACCTTACCGTCGTTTCCTCGGCGTA<br>CTACCACGTTGGCTACCCTGTGAGGTTAGCTTCTGTAGGGATCTA<br>CCCTGCAGAGGTTACCCTGTACGGGGGATGATGCCATGATGTTG<br>GCTACCCTGGAGAAGTAATTCTTCGGGGAAGGATGCCACTGGTA<br>GCGTCGAAAGGACCCATCGAATACTTGTTTCTCTTCCAGTTCCAA<br>TGAGTTAGAATATACCTGATCGAACTTTCTGGGGGCTTTTTCTGG<br>TGGCGGCGCTCGAAAACTTCTACATATAACTGCAAGCTCGAACG<br>TATATAGGTCGATAGTGTGGTGATATGTGTTTTTTTGTAGGCGTGA<br>CTTGTCCGCTCGAAATGTGT
	**TUB2**	GGCAAACCATCTCTGGCGAGCACGGTCTCGACAGCAATGGCGTG<br>TAAGTATATCAATTGTCGATTCACGACGCTAAGAAGCGCAACTGA<br>CGACCAATAAACAGCTACAACGGAACCTCTGAGCTCCAGCTCGA

	ACGCATGAGCGTTTACTTTAACGAGGTACGCAACCGGGGCAACA CCTTACATTGGTTTAAGAGTAGTTACTAATCACCCCAACATGCACA GGCATCTGGTAACAAGTATGTTCCCCGAGCCGTCCTCGTCGATCT CGAGCCGGGTACCATGGACGCCGTCCGTGCCGGCCCCTTCGGCC AACTTTTCCGTCCCGACAACTTCGTCTTCGGCCAGTCTGGTGCCG GAAACAACTG

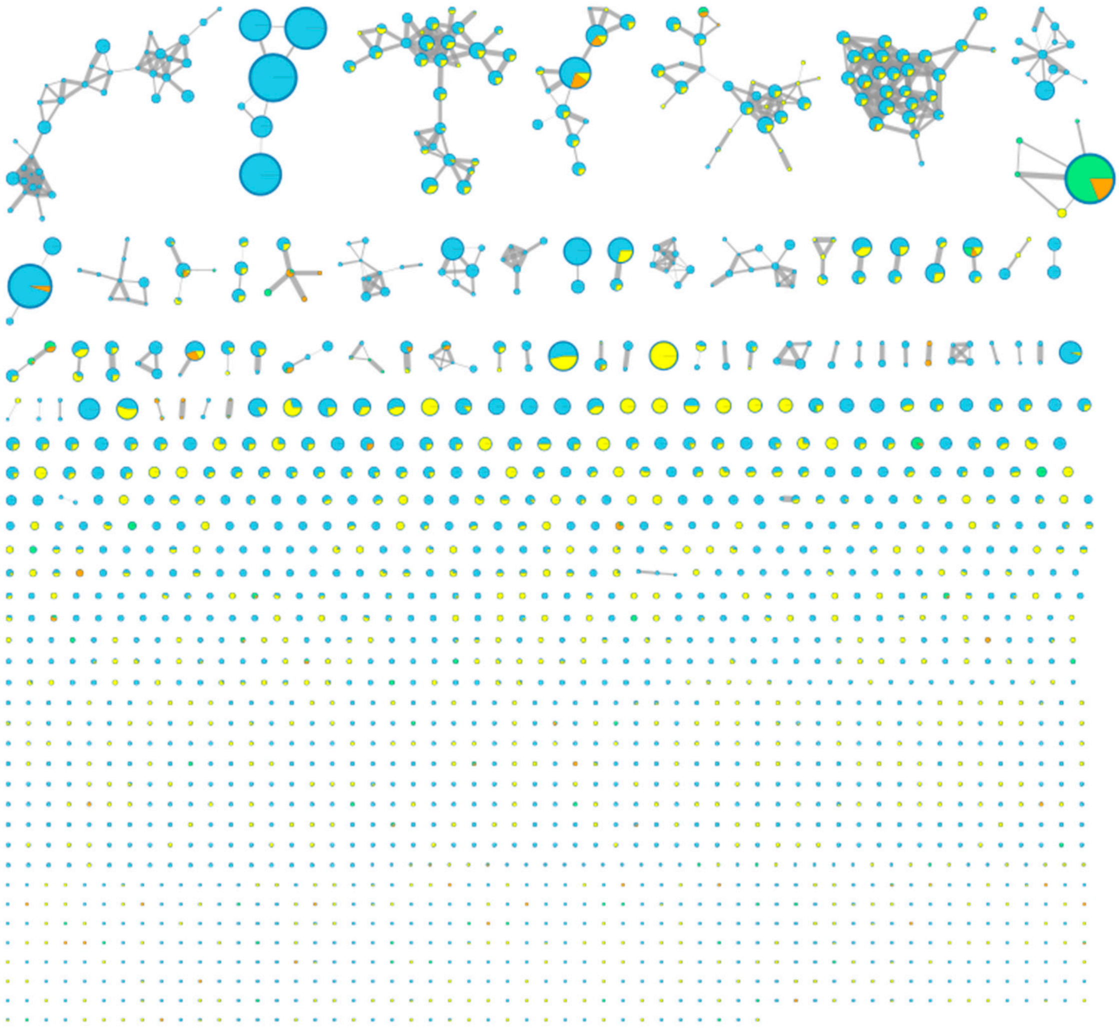

Figure S3A. *Molecular networking of the crude extracts of endophytic fungi isolated from Calea pinnatifida.*

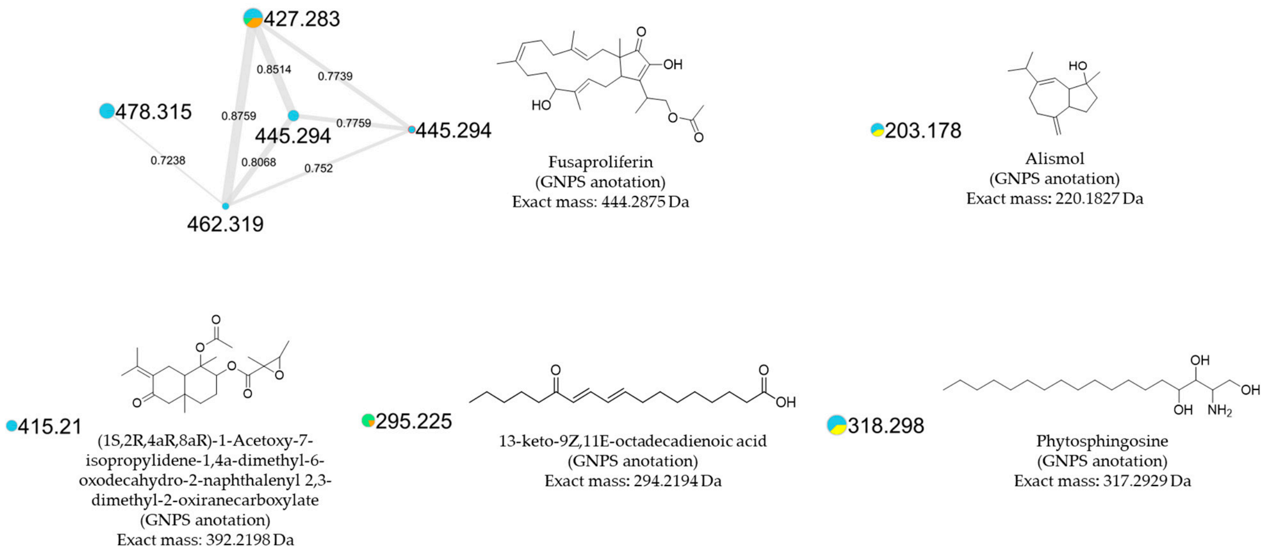

Figure S3B. *Molecular networking of the crude extracts of endophytic fungi isolated from Calea pinnatifida.*

Figure S4. *NMR spectra of the isolated compounds.*

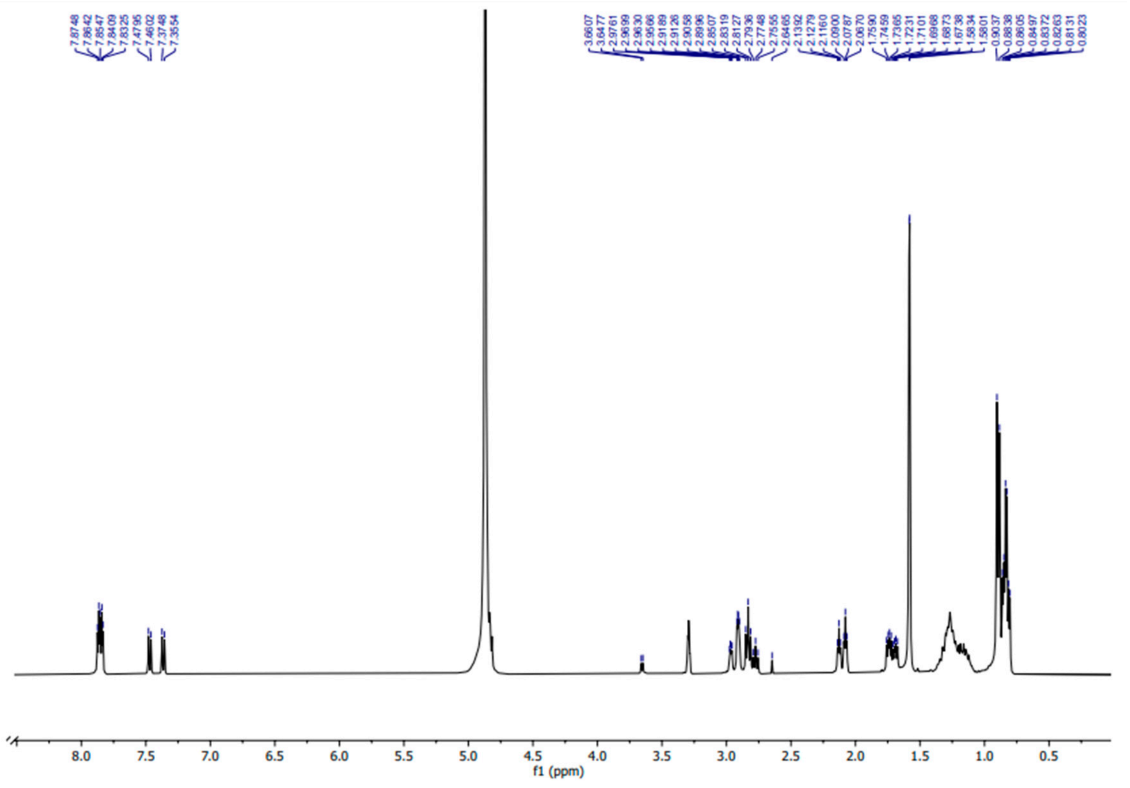

Figure S4A. ^{1}H NMR spectrum of Nodulisporacid A (isomers) 300 MHz, MeOD-d_4.

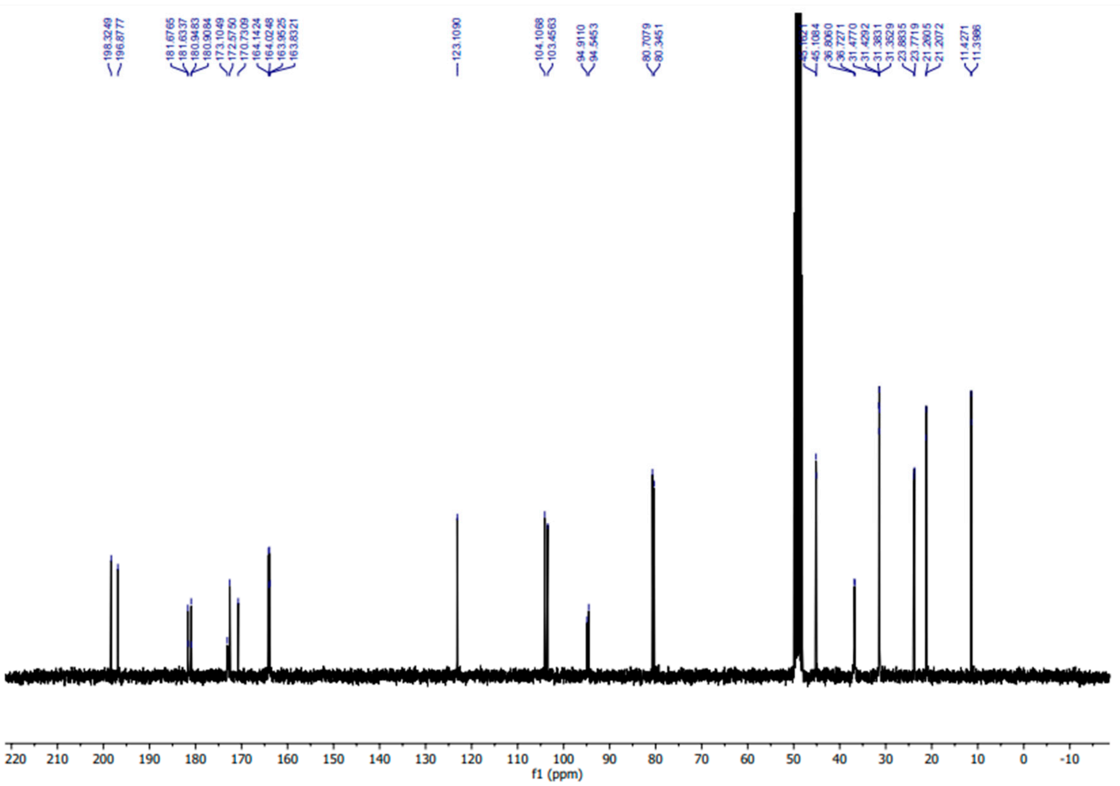

Figure S4B. ^{13}C NMR spectrum of Nodulisporacid A (*E*/*Z* isomers) 300 MHz, MeOD-d$_4$.

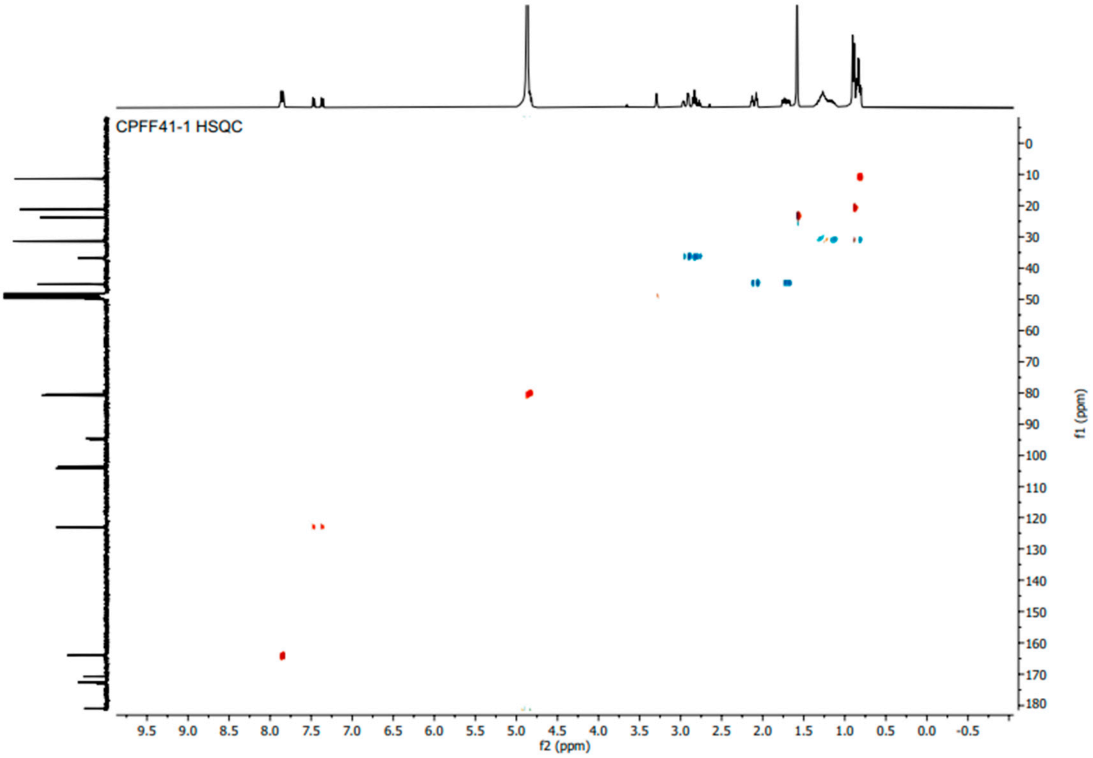

Figure S4C. HSQC NMR spectrum of Nodulisporacid A (*E*/*Z* isomers) 300 MHz, MeOD-d$_4$.

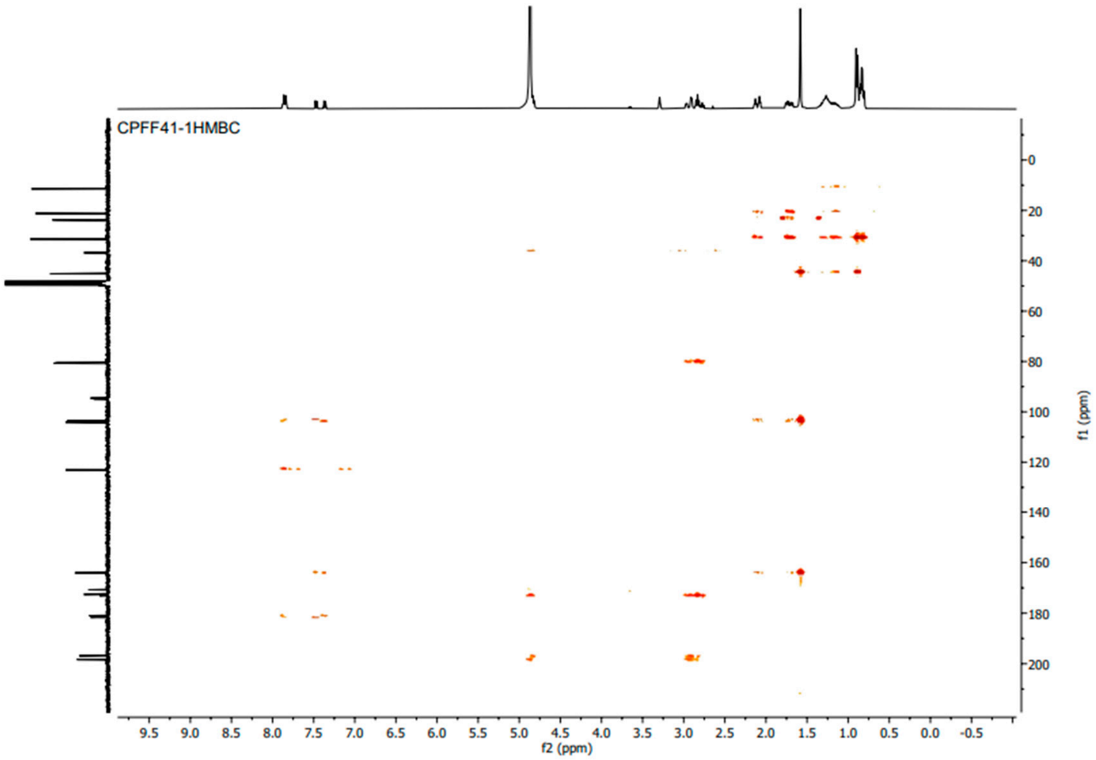

Figure S4D. HMBC NMR spectrum of Nodulisporacid A (*E*/*Z* isomers) 300 MHz, MeOD-d4.

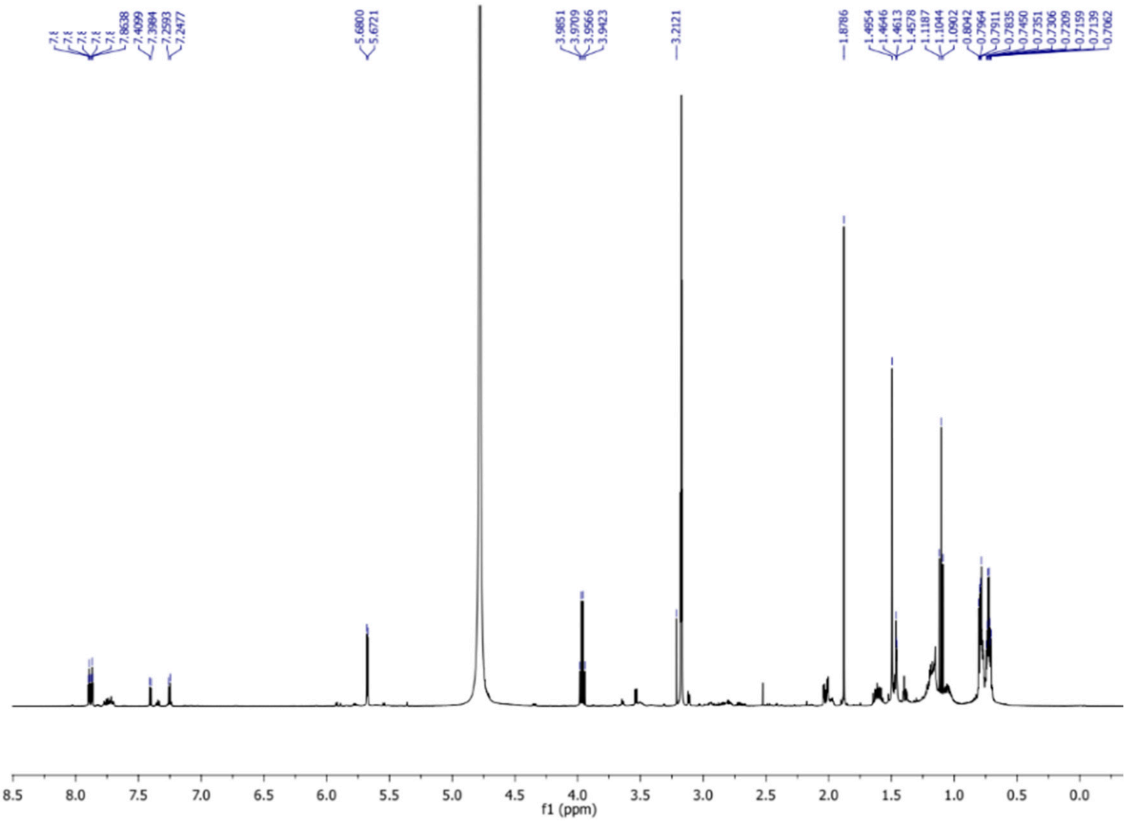

Figure S4E. ^{1}H NMR spectrum of Nodulisfuranol (*E*/*Z* isomers) 300 MHz, MeOD-d4.

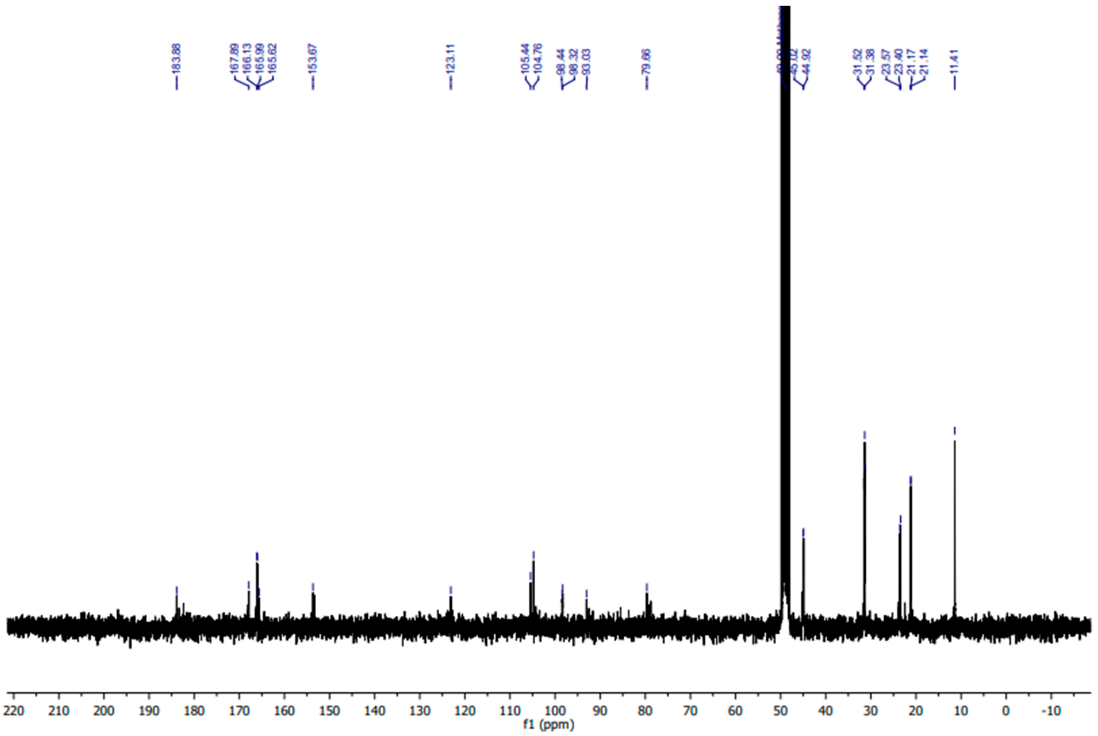

Figure S4F. ^{13}C NMR spectrum of Nodulisfuranol (*E*/*Z* isomers) 300 MHz, MeOD-d_4

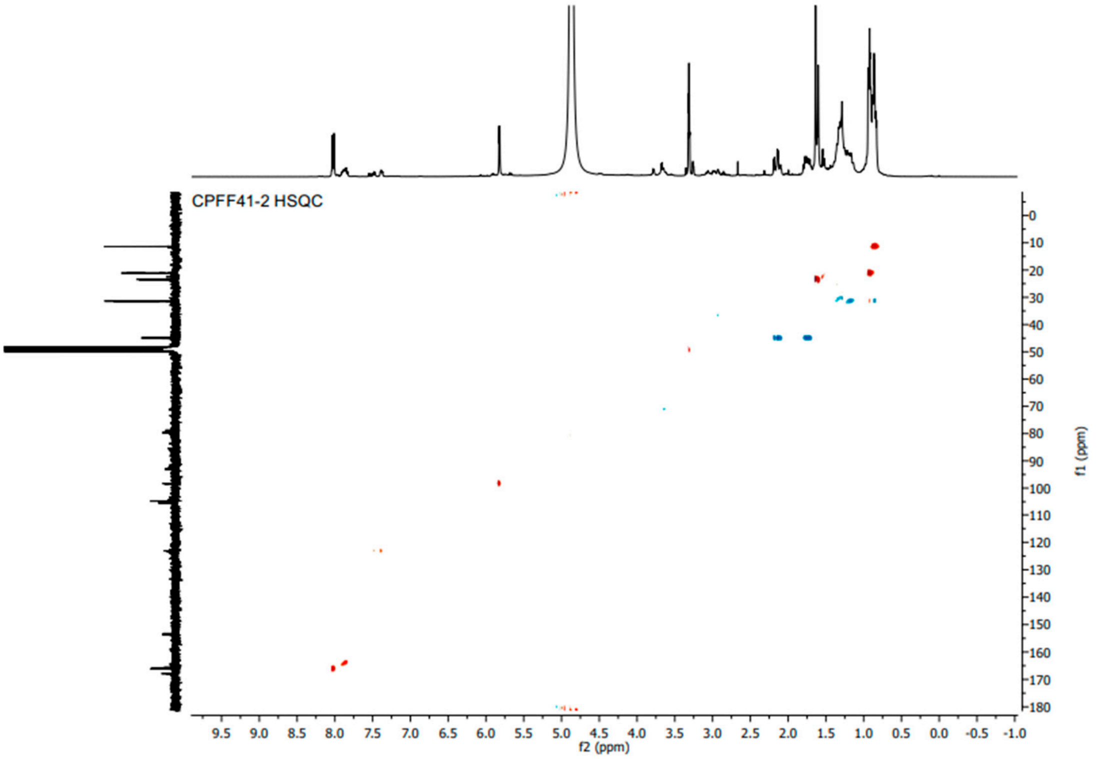

Figure S4G. HSQC NMR spectrum of Nodulisfuranol (*E*/*Z* isomers) 300 MHz, MeOD-d_4.

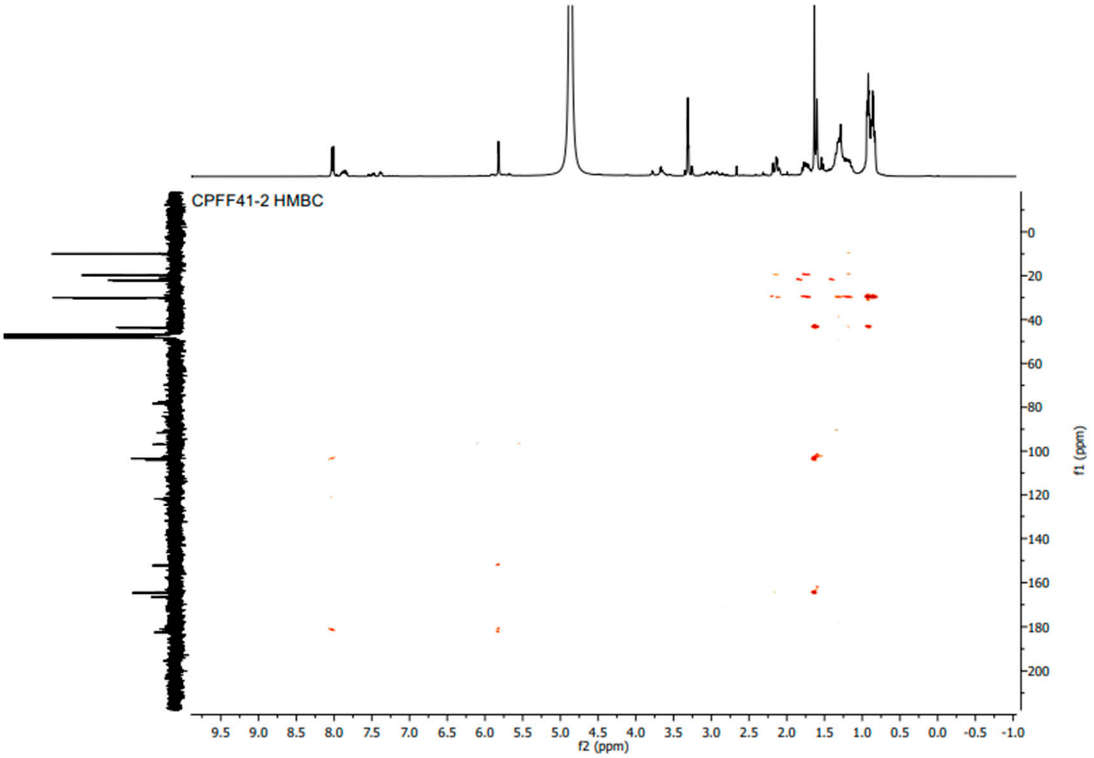

Figure S4H. HMBC NMR spectrum of Nodulisfuranol (*E*/*Z* isomers) 300 MHz, MeOD-d$_4$.
